# Exploring proteins within the coccolith matrix

**DOI:** 10.1038/s41598-024-83052-9

**Published:** 2024-12-30

**Authors:** Craig J. Dedman, Nishant Chauhan, Alba González-Lanchas, Chloë Baldreki, Adam A. Dowle, Tony R. Larson, Renee B. Y. Lee, Rosalind E. M. Rickaby

**Affiliations:** 1 Department of Earth Sciences, University of Oxford, South Parks Rd, Oxford, OX1 3AN UK; 2 School of Geography, Earth and Environmental Sciences, Portland Square, University of Plymouth, Plymouth, PL4 8AA UK; 3 Department of Earth Sciences, University of Cambridge, Downing Street, Cambridge, CB2 3EQ UK; 4 Department of Biology, Bioscience Technology Facility, University of York, York, UK; 5 School of Biological Sciences, University of Reading, Whiteknights, Reading, RG6 6UB UK

**Keywords:** Biomineralisation, Phytoplankton, Calcification, Coccolithophore, Carbon cycle, Cell biology, Microbiology, Molecular biology, Plant sciences, Biogeochemistry, Ocean sciences

## Abstract

Coccolithophores comprise a major component of the oceanic carbon cycle. These unicellular algae produce ornate structures made of calcium carbonate, termed coccoliths, representing ~ 50% of calcite production in the open ocean. The exact molecular mechanisms which direct and control coccolith formation are unknown. In this study, we report on the presence and functional features of proteins within the coccoliths produced by a range of model coccolithophore species including: the globally abundant and well-studied *Gephyrocapsa huxleyi* (formerly *Emiliania huxleyi*) and related *Gephyrocapsa oceanica*, as well as the larger and more heavily calcified *Coccolithus braarudii*. Protein features were compared between species and against biomineralisation proteins previously identified in other marine calcifying organisms. Notably, several protein features were consistently seen across the examined coccolithophore species, including the cell signalling 14-3-3 domain, chromosome segregation SMC ATPase domain, as well as proteins involved in protein processing and protease inhibition. The copper-binding cupredoxin domain was observed in both *Gephyrocapsa* species, as well as other marine calcifiers, suggestive of a requirement of Cu in biomineralisation. Building consensus with existing work, we highlight the pentapeptide repeat as a feature which is associated with the coccolith matrix, being identified in all three examined species, and propose that this structural motif may play a role in controlling coccolith growth. This preliminary study provides insight towards the functional diversity of calcification machinery in coccolithophores and presents a number of candidates for future research towards understanding the biochemical controls which direct coccolithogenesis.

**Supplementary Information:**

The online version contains supplementary material available at 10.1038/s41598-024-83052-9.

## Introduction

The marine calcifying phytoplankton, coccolithophores, produce ornate scales made of calcium carbonate (CaCO_3_) which, upon secretion, form an intricate coccosphere on the cell surface^1^. These mineralised scales, known as coccoliths, play a key role in the long-term marine carbon sink, whereby ~ 50% of the overall rain of calcite to the seafloor is made up of these biologically mineralised particles^2–4^. Given their calcifying and photosynthetic activities, coccolithophores play a critical role in marine ecology and biogeochemistry, and the advent of this biomineral flux to deep sea sediments has provided buffering of ocean carbonate chemistry for the last 200 Myrs, influencing global climate^1^. The relative abundance and preservation of fossil coccoliths (i.e., nannofossils) within sediments of the sea floor, has facilitated their use as an indicator and bearer of geochemical proxy information for paleoclimatic reconstruction^5–8^, and provide further insight into evolutionary processes^9,10^.

Owing to the ecological and biogeochemical importance of calcification by coccolithophores, significant research efforts have been directed at establishing the exact biological mechanisms by which coccoliths are made (coccolithogenesis) and excreted at the cell surface e.g.,(^1^). Such knowledge has implications for understanding the environmental parameters involved in its inception and activity, as well as explaining the likely role of calcification in the cell and ecosystem^11^. A general model for the structural controls on coccolithogenesis has been proposed, based largely on electron microscopy, however the molecular mechanisms which drive this process remain largely unclear^9,12–15^. Such work is limited to a select group of species for which laboratory culturing is achievable, most commonly *Pleurochrysis* (*Chrysotila*) *carterae*, *Coccolithus sp.* and the globally-abundant *Gephyrocapsa huxleyi* (formerly *Emiliania huxleyi*)^9,12–15^. Coccolithogenesis in many taxa, including those studied here, takes place within a specialised coccolith vesicle (CV) derived from the Golgi apparatus^11,16^. In close proximity to the CV, a reticular body is proposed to supply essential calcification substrates i.e., Ca^2+^ and HCO_3_^−^, maintaining supersaturated conditions at the site of mineralisation^17^. Meanwhile to ensure conditions remain favourable for CaCO_3_ formation, protons must be actively removed from the CV, requiring strict control^18^. To transport the coccolith to the cell surface, and potentially to provide a mould for its morphology^14,19–24^, the cytoskeletal system must play an important role. Inhibition of normal cytoskeletal function is reported to result in malformation of the coccolith structure^25^, however it is unclear as to whether this is due cellular stress or a direct impact on coccolithogenesis.

The biomineral calcite of calcifying organisms can comprise up to 5 wt% of organic molecules^26^. These compounds represent polysaccharides and proteins which are embedded into the calcite matrix. Although just a small fraction of overall calcite weight, research points to a key role of these organic molecules in the process of calcification^26^. Understanding the organic components of coccoliths, and their localisation, will undoubtedly enhance our understanding of the process of biomineralisation in these species^27^. Specifically in coccolithophores, coccolith-associated polysaccharides (CAPs) have been identified to potentially regulate crystal growth and remain attached to coccoliths upon secretion^28,29^. Variations are observed in the estimated costs of CAP production in various species, representing 7% of photosynthetically fixed carbon in *G. huxleyi*, compared to just 0.2% in the larger more heavily calcified *Coccolithus braarudii*, possibly indicating a varying role of CAPs in these different species and competition for internal resources^29^. To date, CAPs from only a few species have been characterised to limited detail^30,31^. Even less is known on the structure and function of proteins held within the coccolith matrix and how these may interact with CAPs. Such proteins may act as nucleators, scaffolds, transporters of calcification substrates, or as suppressors of crystal growth^32–35^. Alternatively, proteins may function to modify other proteins, or molecular components such as CAPs in the calcification pathway. Uncovering the presence and molecular function of proteins held within the CV and growing coccolith is key to gaining a complete understanding of the process of coccolithogenesis in coccolithophores.

By examining the proteins associated with calcium carbonate structures such as the shell or skeletal matrix, a number of calcification-related proteins have been identified in marine calcifiers such as bivalves, echinoderms and corals. For example, a core set of > 100 proteins have been identified in the coral skeletal organic matrix (SOM)^36–39^. It is possible that in coordination with CAPs, species-specific variations in coccolith-protein structure and abundance may play a part in determining overall coccolith morphology which shows great variation across coccolithophore taxa and even among strains. For example, by varying the enantiomers of chiral amino acids it is possible to alter the spiralling morphology of CaCO_3_ (vaterite) from a clockwise to anti-clockwise direction^40^. By altering pH of the reaction, this spiral morphology can be altered further^40^, highlighting the impact that subtle changes to protein structure and biochemical control of the composition at the site of biomineralisation can have on calcite crystal morphology and relationships with neighbours. Such biochemical variation may have increased diversity between coccolithophores through evolution. Although, it is important to clarify that extant organisms exclusively produce the L-enantiomer of chiral amino acids^41^, thus a change in enantiomer unlikely contributes to evolution of varied morphology, but demonstrates the impact of specific biochemical conditions on regulation of crystal growth. Identification of conserved functional protein domains may shed light on the likely processes which gave rise to and may have been repurposed for the inception of calcification in these species. Previous works, for example those focussing on CAP extraction as well as characterisation of coccolith calcite by X-ray powder diffraction, suggested that polysaccharides were the primary macromolecular component and that proteins were likely not incorporated into the coccolith matrix^42–44^. To our knowledge, just one published study exists, confirming the presence of proteins within the coccoliths of *G. huxleyi*^45^. In this comprehensive study Skeffington et al. (2023) present an important update on the genome of *G. huxleyi*, the keystone model of coccolithophore research^12^, and explore the evolutionary history of encoded proteins. Subsequently, these protein sequences were utilised as a reference for proteomic analyses of various cellular compartments involved in coccolithogenesis including the CV and coccosphere, revealing candidate proteins potentially involved in this process^45^. In total, 26 proposed coccolith proteins were identified in this previous work, where the pentapeptide-repeat domain was a common feature, found in six proteins^45^. Notably, in this work a significantly higher mass of coccolith calcite was required for protein extraction compared to that previously used for CAPs^42,45^, perhaps explaining the lack of identification of proteins in previous work.

To further explore the presence and possible roles of proteins incorporated within the coccolith matrix, we have carried out a preliminary investigation upon three widely used coccolithophore model species; the globally dominant *G. huxleyi* and closely related *Gephyrocapsa oceanica*, as well as *Coccolithus braarudii*, representing the Isochrysidales and Coccolithales orders, respectively. Each species was maintained in batch culture in the laboratory and coccolith material harvested for proteomic analysis. This work provides an advance in our knowledge on the existence of proteins within the coccolith matrix, and importantly further suggests that the pentapeptide repeat represents a structural protein domain which is conserved across coccoliths of each species, potentially playing a key role in the calcification pathway.

## Methods

### Coccolithophore culture

**Fig. 1:**
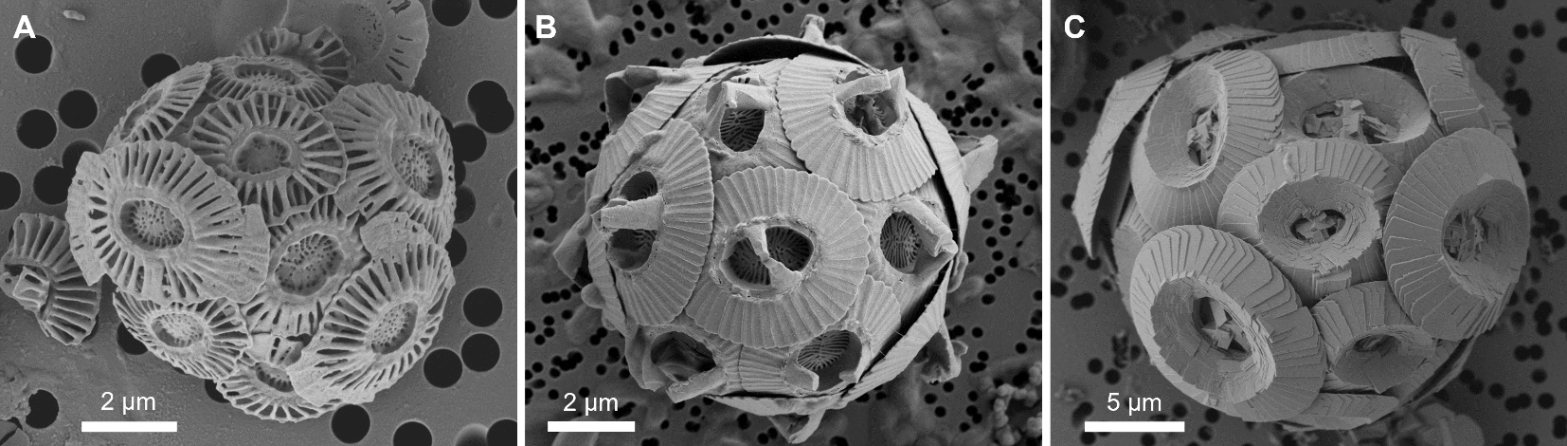
Scanning electron microscope images of the coccolithophore strains used in this study; (**A**) RCC1731 *Gephyrocapsa huxleyi*, (**B**) RCC1314 *Gephyrocapsa oceanica*, (**C**) RCC1198 *Coccolithus braarudii*.

Clonal cultures of *Gephyrocapsa huxleyi* (RCC1731 and non-calcifying RCC1242), *Gephyrocapsa oceanica* (RCC1314) and *Coccolithus braarudii* (RCC1198) (Fig. 1) were obtained from the Roscoff Culture Collection (Roscoff, France). These species were selected owing to their use as key models in coccolithophore research and based on availability of genomic or transcriptomic data for generation of peptide sequence databases for protein identification. Additionally, these species represent members of the Isochrysidales and Coccolithales orders, respectively^46^. A non-calcifying strain was selected as a control to assess contamination of calcite by cellular material and effectiveness of the cleaning protocol, outlined in detail in Section “Collection & cleaning of coccoliths”. Cultures were maintained in synthetic ocean water buffered using 1 mM Tris, supplemented with modified K/2 nutrients^47–49^. To avoid bacterial contamination, all media was filter-sterilised (0.22 μm) prior to use and cultures were monitored by microscopy and flow cytometry to ensure presence of monocultures. All cultures were maintained in a PHCbi MLR-352 Climate Chamber (17 °C) at a light intensity of 60–80 mol m^−2^ s^−1^ under a 14:10 h light: dark cycle. Each species was maintained in the exponential growth phase by transfer of cells into fresh growth media within semi-continuous batch cultures. For *G. huxleyi* and *G. oceanica*, cell densities were maintained between 0.5 and 2.0 × 10^6^ cells mL^−1^. For *C. braarudii*, the cell densities were kept between 0.5 and 2.0 × 10^5^ cells mL^−1^.

### Collection & cleaning of coccoliths

Coccolith calcite was collected six hours into the light cycle by centrifugation of culture biomass (4700 rpm; 20 min) and stored at -20 °C. To ensure sufficient material for effective protein extraction and analysis, coccolith material was pooled from a total of 50 L of semi-continuous batch culture, providing one analytical sample for each species. The resultant coccolith calcite pellets derived from each species were first cleaned with 50% NaOCl (Bleach, 12%) and 50% H_2_O_2_ (30%). The pH of the cleaning solution was buffered with NaOH to be alkaline (> pH 8) to minimise calcite dissolution. After addition of the cleaning solution, pellets were gently vortexed and kept in the dark overnight at 5 °C. The following day, samples were centrifuged (3000 rpm; 15 min), washed twice with 50 mM CaCl_2_ (pH 9) and resuspended in cleaning solution. This process was repeated five times and the presence/absence of residual organic matter inspected by eye. Following this, calcite pellets were cleaned in 30% H_2_O_2_ (pH 8) in the dark, overnight three times to eliminate any surface adhering organics. In addition, calcite pellets were cleaned with a solution containing 6% NaOCl and 1% (v/v) Triton-X 100 buffered with 0.05 M NH_4_HCO_3_. During this cleaning step, the pellets were kept on a rotating shaker at 30 rpm overnight. This was repeated three times. The final cleaning phase involved centrifugation of calcite pellets (3000 rpm; 15 min), washing using 50 mM CaCl_2_ (pH 9) three times and centrifugation at 1500 rpm for 8, 5, and 5 min between the washing steps. The clean pellets were then frozen at -20 °C before decalcification and protein extraction. To measure the efficacy of the cleaning process, and to identify proteins that were resistant to the cleaning process, an inorganic calcite blank (control) was processed in an identical manner as coccolith pellets. This control sample contained 0.1 g of non-calcifying *G. huxleyi* (RCC 1242) biomass and 1 g inorganic calcite.

### Protein extraction & digestion

After cleaning, samples were decalcified in 0.5 M EDTA (pH 8) overnight, centrifuged (4500 rpm; 30 min) to remove particulate matter. The supernatant containing the EDTA-soluble fraction was used for protein extraction using the trichloroacetic acid (TCA) precipitation method^50^. Samples were resuspended in Tris-HCl buffer (pH 8.0) and stored at -20ºC. In-solution trypsin digestion was carried out to digest extracted proteins. Briefly, 50 µg of protein per sample was denatured in 4 M urea in 100 mM ammonium bicarbonate for 10 min at room temperature under shaking (650 rpm). Following this, cysteines were reduced using 10 mM tris(2-carboxyethyl)phosphine (TCEP, Bond-Breaker™ Solution) for 30 min at room temperature, and subsequently alkylated with 50 mM iodoacetamide for a further 30 min in the dark. Proteins were pre-digested using LysC, to avoid missed cleavages of lysine by trypsin alone^51^, and incubated for 2 h at 37 °C under shaking (800 rpm). Urea concentrations were diluted to 2 M and CaCl_2_ added at a final concentration of 2 mM, before trypsin (Promega) was added. The trypsin reaction was carried out for 20 h (37 °C, 800 rpm) and stopped by the addition of 5% formic acid. Peptides were desalted directly after digestion using C18 stage tips and dried overnight using a vacuum centrifuge. Samples were stored at -20 °C prior to analysis by mass spectrometry.

### Mass spectrometry

Peptides were loaded onto EvoTip Pure tips for introduction onto an EvoSep One nanoUPLC system. A pre-set 30 SPD gradient was used with a 15 cm EvoSep C_18_ Endurance column (15 cm x 150 μm x 1.9 μm). The nanoUPLC system was interfaced to a timsTOF HT mass spectrometer (Bruker) with a CaptiveSpray ionisation source. Positive PASEF-DDA, nanoESI-MS and MS^2^ spectra were acquired using Compass HyStar software (version 6.2, Bruker), between 100 and 1700 m/z. Instrument source settings were: capillary voltage, 1600 V; dry gas, 3 L/min; dry temperature, 180 °C. TIMS settings were: 1/K0 0.6–1.6 V.s/cm^2^; ramp time, 100 ms; ramp rate, 9.42 Hz. Data dependent acquisition was performed with 10 PASEF ramps and a total cycle time of 1.17 s. An intensity threshold of 2500 and target intensity of 20,000 were set, with an active exclusion applied for 0.4 min post precursor selection. Collision energy was interpolated between 20 eV at 0.6 V.s/cm^2^ – 59 eV at 1.6 V.s/cm^2^.

### Identification of biomineralisation proteins and compositional analysis

Lists of proteins derived from the calcite/shell matrix of marine calcifying organisms, namely coccolithophores, molluscs and corals were collated from the available literature. Protein sequences were obtained from the NCBI Genbank database or publicly available sequences associated with the studies used. The NCBI Conserved Domain Search tool^52^ was used to identify conserved protein domains within the identified proteins and Expasy’s ProtParam tool used to calculate acidic residue content^53^.

### Analysis

Data analysis was performed using PEAKS Studio 10 (Bioinformatics Solutions Inc.)^54^. To identify proteins in the control and *G. huxleyi* samples, the updated Emihu2 reference proteome was utilised^45^. Owing to the fact that no reference genome exists for either *G. oceanica* or *C. braarudii*, raw spectra were searched against the NCBI subset of proteins for each species (accessed 28/06/24) appended with common proteomics contaminants. Database searching was conducted using PEAKS *de novo*, database searching, PTM and Spider modules, with the following parameters: MS^1^ error tolerance, 15.0 ppm; MS^2^ error tolerance, 0.05 Da; precursor mass, monoisotopic; enzyme, trypsin; max missed cleavages, 1; digest mode, specific; peptide length range, 6–45; fixed modifications, beta-methylthiolation (+ 45.99); variable modifications, oxidation (M) (+ 15.99), max variable PTM per peptide, 2; *de novo* matches are non-probabilistic and filtered to 50% ALC scores. PEAKS PTM searching included consideration of 313 common variable modifications within identified proteins and the Spider module incorporated single point amino acid substitutions. PEAKS peptide matches are filtered to 1% peptide spectrum match (PSM) false discovery rate (FDR) as assessed empirically against a decoy database search. To improve accuracy, proteins taken forward for analysis were identified based on ≥ 2 peptides. Proteins identified as common proteomics contaminants were removed from analysis, as well as any protein identified in the control. To identify conserved domains and explore structural features of identified coccolith proteins, sequences were submitted to the NCBI CD-Search and InterProScan web-based tools^52,55,56^. Protein amino acid composition was determined using the ProtParam tool on the Expasy server^53^. Here, the protein with highest coverage was selected from each protein group identified and analysed using the ProtParam tool on the Expasy server to determine % acidity, based on the presence of aspartic and glutamic acid residues. Where proteins displayed equal coverage, average values were obtained. The NCBI BLASTp tool was utilised to assess sequence similarity between proteins identified in each species^57^.

## Results and discussion

**Fig. 2:**
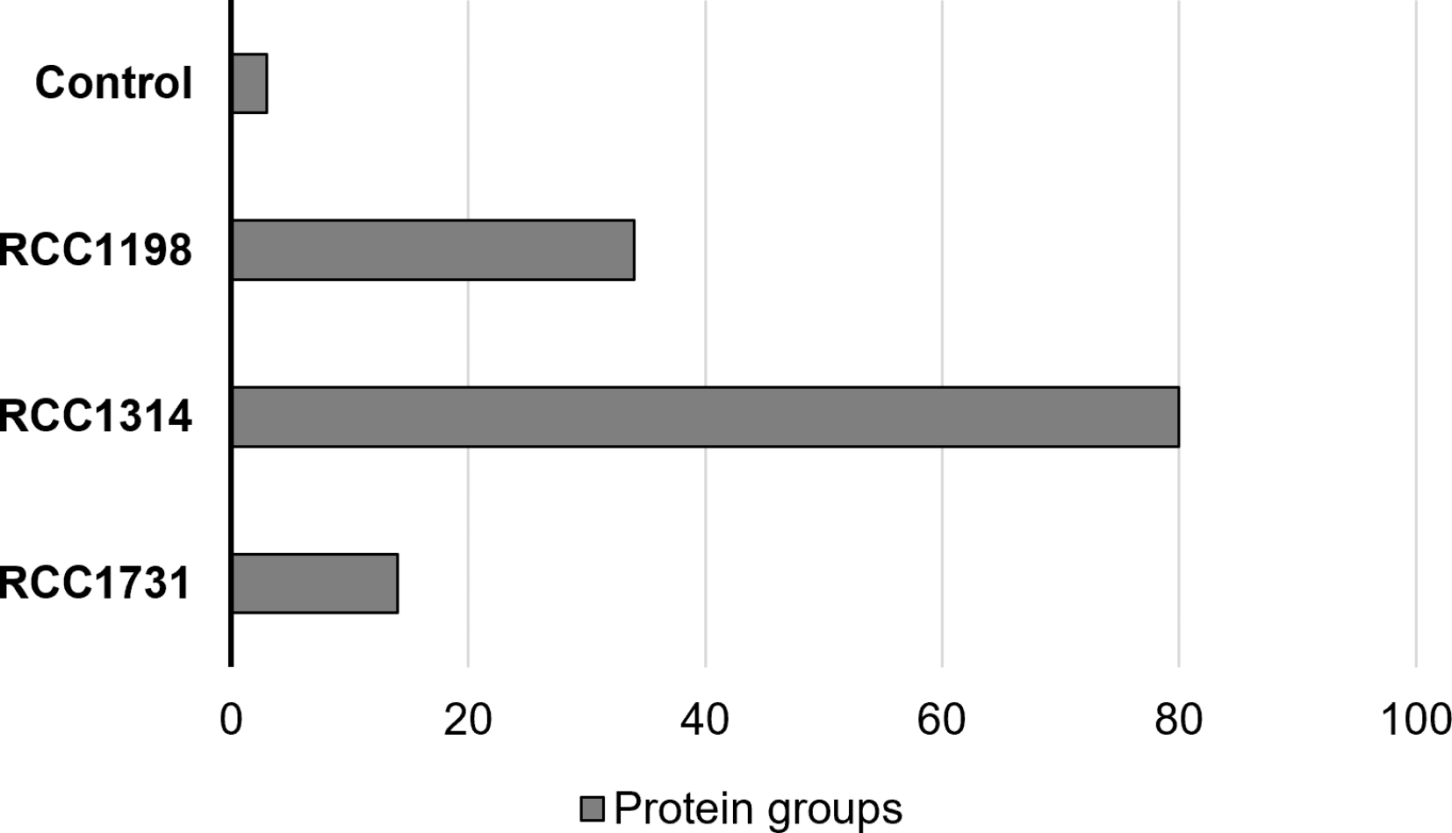
Number of protein groups identified in each species: RCC1731 *G. huxleyi*; RCC1314 *G. oceanica;* RCC1198 *C. braarudii.*

Data was filtered to remove common proteomics contaminants and proteins whose identification was based on only one peptide to reduce uncertainty. Notably, a considerable number of contaminants associated with the handling of samples e.g., human keratin, were identified in datasets for each species. After filtering, a total of 3, 14, 80 and 34 protein groups (≥ 2 peptides), were identified in the control, *G. huxleyi*, *G. oceanica* and *C. braarudii* samples, respectively (Fig. 2). Proteins identified in the control (histone H4 and heat-shock protein 70) were removed from downstream analyses of each species of interest, owing to the fact these could represent cellular contamination. However, both heat-shock proteins HSP70 and HSP90 have previously been recorded in *G. huxleyi* proto-coccolith fractions^45^. Following their removal, a total of 13, 79 and 29 proteins were considered as potential coccolith proteins for further analysis in *G. huxleyi*,* G. oceanica* and *C.* braarudii, respectively (Tables SI.1–4). Protein sequence coverage ranged from 1 to 58% (Supplementary Data Table 1). The number of identified protein groups varied considerably between the two *Gephyrocapsa sp.*. For *G. oceanica* the number obtained is comparable to that observed in previous studies, but our harvest of proteins appears considerably reduced for *G. huxleyi* compared to published data^45^, and may be due to differences in the method used for coccolith isolation. The relatively lower number of protein groups identified in *C. braarudii* compared to *G. oceanica* is likely due to identification challenges rather than harvest yield due to the limited availability of *Coccolithus sp.* protein sequences currently in the NCBI database. However, this dataset remains of high utility given that no published proteomic data currently exists for this species.

### Functional analysis of potential coccolith proteins

Owing to the limited functional annotation associated with the proteins identified, the NCBI conserved domain search (CD-Search) and InterProScan tools were used to identify protein features and consider any possible role of identified proteins in the coccolithogenesis pathway^52,55,56^. Overall, significant hits for CD-Search were recorded for ~ 80% of protein groups across the three species, while InterProScan was used to provide additional functional insight, including predicted Gene Ontology and the presence of signal peptide regions (Supplementary Data Table 1 and SI.1–4). It has been suggested that proteins localised to the CV should contain a signal peptide region, facilitating their import into the ER and transport via the Golgi to the CV^45^. In our work, 8/13, 36/79 and 3/29 of protein groups contained at least one protein sequence predicted to contain a signal peptide in *G. huxleyi*,* G. oceanica* and *C. braarudii*, respectively (Tables SI.1–4). Whilst previous work restricted candidate coccolith-associated proteins to only those containing signal sequences^45^, herein we consider all identified proteins, highlighting those with relevant structural features which point to a possible role in the calcification pathway but using caution when considering proteins which may derive from cellular material. It is possible such signal regions may simply not be present in the reference protein sequences used in the study, particularly those for *G. oceanica* and *C. braarudii* for which no reference genome currently exists and where only partial protein sequences are available for some proteins.

A total of 13 protein groups, were identified within the *G. huxleyi* dataset (Table SI.2). Protein coverage ranged from 1 to 30%. Four protein groups were made-up of pentapeptide-repeat containing proteins, of which two possessed a signal peptide region. Protein groups containing actin, cupredoxin, chromosome segregation ATPase, and 14-3-3 family proteins were also identified. Four protein groups contained no conserved domain, however all proteins derived from these groups possessed a signal peptide region. Despite a reduced protein yield, 6 of the 26 proposed coccolith proteins reported by Skeffington et al. (2023) were identified in our *G. huxleyi* dataset, representing a sizeable fraction of the proposed candidate coccolith proteins in this work. These overlapping proteins are displayed in Table SI.5 and include two pentapeptide-repeat containing proteins, as well as four with no conserved protein domain. Each of these proteins were predicted to contain a signal peptide region, possibly locating them to the CV. Considering the results of the phylostratigraphic analysis conducted by Skeffington et al. (2023), three of the overlapping proteins were predicted to be of calcihaptophyta age, whilst the remaining three were specific to the Gephyrocapsa genus, including the two overlapping pentapeptide-repeat containing proteins identified^45^. Hence, these proteins represent suitable candidates for future research on the biochemical control of coccolithogenesis in *G. huxleyi* and exploration of any strain-dependent variation in gene or protein sequence which may influence calcification morphology and intensity.

The largest number of protein groups (79) was identified in *G. oceanica* (Table SI.3). Here, protein coverage ranged from 4 to 58%. Thirty protein groups (~ 38%) were made-up of pentapeptide-repeat containing proteins, a third of which contained a signal peptide region. Almost half of *G. oceanica* protein groups (36) contained a signal peptide region. Four protein degradation-related domain containing groups were identified, as well as one peptidase inhibitor family. One heat shock protein 90 family protein was recorded, and four groups contained conserved domains relating to cell signalling or protein-protein interaction. An additional four protein groups possessed domains relating to carbohydrate metabolism, three relating to methyltransferase activity, and 18 had no conserved domain present, nine of which contained a signal peptide region.

Twenty-nine proteins were identified in *C. braarudii* coccoliths, with coverage ranging 5–32% (Table SI.4). Here, two histone family protein groups were identified, as well as two relating to RNA-binding, translation and helicase activity, respectively. A total of six protein groups contained conserved domains with functions relating to central energy and carbon metabolism likely representing cellular contamination, including two ATP synthase and two glyceraldehyde-3-phosphate dehydrogenase domains. Two pentapeptide-repeat protein groups were identified, as well as four protein groups with no conserved domain. Only three protein groups identified in *C. braarudii* contained a signal peptide region.

Overlapping functional aspects of coccolith proteins identified in this study are presented in Table 1. To aid our understanding of the potential role of identified coccolith proteins in the calcification pathway and biomineralisation in general, previously reported protein sequences associated with the calcite/shell matrix of marine calcifying organisms, i.e., corals and molluscs, were collated and examined in terms of the functional domains present and amino acid composition. We identified 161 calcite/shell matrix proteins from work on marine molluscs, corals and the coccolithophore *G. huxleyi* (Supplementary Data Table 2). In accordance with the identification of the pentapeptide repeat in coccolithophores (this study and Skeffington et al.^45^), repetitive domains appear a common feature of both mollusc and coral proteins. For example, the cadherin tandem repeat domain is present in a number of coral skeletal matrix proteins^36,37^. Likewise, the presence of proteins involved in protein modification e.g., ubiquitin, and proteolysis are also consistently reported in coral species^36,37^.

**Table 1:** Conserved features of coccolith proteins identified across species.

Protein feature	RCC1731 G. huxleyi	RCC1314 G. oceanica	RCC1198 C. braarudii	Identified in other marine calcifiers
14-3-3 protein domain	Yes (1)	Yes (1)	Yes (1)	
ATP synthase		Yes (1)	Yes (2)	
Chromosome segregation ATPase Smc	Yes (1)		Yes (1)	Yes^36^ (*Stylophora pistillata*)
Cupredoxin	Yes (1)	Yes (1)		Yes^37^ (*Acropora millepora*)
DNA/RNA-related, including; transcription/translation	Yes (1)	Yes (2)	Yes (6)	Yes^58,59^ (*Pinctada fucata; Pinctada maxima*)
P-loop containing nucleoside hydrolase domain		Yes (1)	Yes (1)	
Pentapeptide-repeat containing protein	Yes (4)	Yes (30)	Yes (2)	Yes^45^ (*Gephyrocapsa huxleyi*)
Protein transport		Yes (1)	Yes (1)	
Proteolytic domain		Yes (4)	Yes (1)	Yes^37^ (*Acropora millepora*)
Ubiquitin & ubiquitin-like		Yes (2)	Yes (1)	Yes^36^ (*Stylophora pistillata*)
No conserved domain	Yes (4)	Yes (18)	Yes (4)	Yes^36,37,45,58–70^ (Multiple)

*Number of protein groups containing each conserved feature is presented in brackets for each species. In the right-hand column, the marine calcifying species associated with each feature is listed.

The copper binding cupredoxin domain which typically functions in electron transport, for example in the plastocyanin family proteins, was identified in both *G. huxleyi* and *G. oceanica* in our study. This domain is present in the hephaestin-like protein of the coral *Acropora millepora*^37^, with multicopper oxidase (MCO) proteins being conserved in the skeletons of both scleractinian and octocoral species^71^. Hephaestin-like proteins have also been identified in the shells of brachiopods and are reported to be involved in the formation of radula^72,73^. It has been proposed that MCOs may have developed a specific function in biomineralisation within certain lineages^71^, therefore its presence in coccoliths in this study is of high interest as it suggests a wider conservation of such domains in biomineralisers across animal and plant kingdoms.

Whilst its function in calcification may not be immediately apparent, the chromosome segregation ATPase SMC domain was observed in both *G. huxleyi* and *C. braarudii* in this study. This domain has previously been recorded in protein derived from the skeleton of the coral *Stylophora pistillata*^36^. Several other proteins possessing domains which typically function in DNA and RNA-related processes were also identified in the examined coccolithophore species.

Unlike proteins derived from molluscs and corals, we did not identify carbonic anhydrase in our work, nor was this protein identified in previous work on *G. huxleyi* coccoliths^45^. Rather, this protein has been associated with the coccosphere of *G. huxleyi*^45^ and this difference may stem from the unicellular nature of coccolithophores and hence lack of direct contact between the site of calcification and extracellular environment. Similarly, the von Willebrand factor A domain was consistently present across a wide range of the mollusc and coral protein sequences identified^36,37,66,74–77^, however, this ligand-binding domain was not observed in the coccolith proteins recorded in this study or previous work^45^. Recent work examining the von Willebrand factor A domain in greater detail within mollusc species suggests that this domain likely contributes to organic complex formation during shell mineralisation^78^.

### Protein acidity

**Fig. 3:**
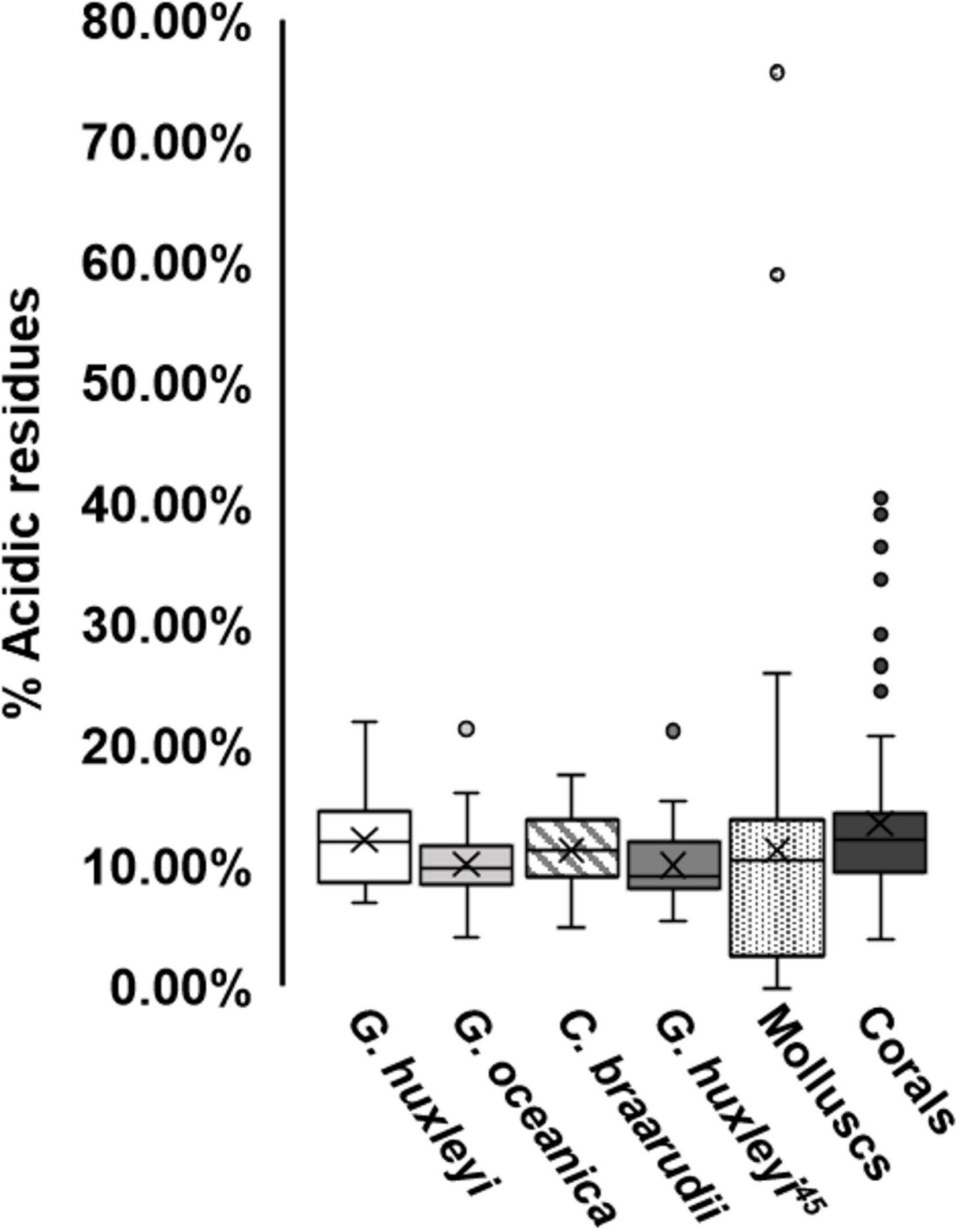
Comparison of the proportion of acidic residues present in identified coccolith proteins and those derived from calcite/shell matrix of other marine calcifying organisms (Supplementary Data Table 2).

Acidic proteins have been implicated in biomineralisation due to their affinity for Ca^2+^ binding, and ability to interact with the growing calcite crystal^33,37,79,80^. Proteins rich in aspartic and/or glutamic acid have a high capacity for Ca^2+^ binding and accumulation, but low specificity^81^. Hence, such acid-rich proteins may function in the formation of Ca^2+^ aggregates^79^. The Expasy ProtParam Tool was utilised to estimate protein acidity (% acidity) of identified calcite matrix proteins by determining the proportion of aspartic and glutamic acid residues^53^. Overall, the average proportion of acidic residues within potential coccolith proteins identified in each of the studied species was relatively consistent, being 12.31 ± 4.30%, 10.21 ± 2.69% and 11.40 ± 3.23% for *G. huxleyi*,* G. oceanica* and *C. braarudii*, respectively (Fig. 3). It must be noted that for a number of proteins, only partial sequences were available for analysis, thus values represent estimates rather than defined values. The five protein groups displaying greatest % acidity in each species are displayed in Table 2. Of note, no protein was predicted to contain a conserved Ca^2+^-binding domain. In each species, 14-3-3 proteins contained relatively high acidic amino acid content, which is a characteristic feature of this domain^82^.

Upon comparison with previously identified calcite matrix proteins (Fig. 3), it is noticeable that the proteins identified in this study display a similar acidic profile to those previously identified in *G. huxleyi* coccoliths^45^. Average % acidic residues were highest in coral sequences, with coral and mollusc proteins displaying greatest variation. A number of coral proteins were identified with acidic residues making up 30–45% of the protein, while two exceptionally acidic aspein proteins (59% and 79%), capable of calcite polymorph regulation^79^, were identified in the pearl oyster species *Pinctada fucata* and *Pinctada maxima*, respectively^65,70^. This difference in % acidity of proteins may arise due to the fact that corals and mollusc species interact with Ca^2+^ in seawater directly, whereas the Ca^2+^ utilised for coccolithogenesis is first filtered by the cell membrane.

**Table 2:** Proteins containing highest % acidity as determined by the Expasy ProtParam Tool.

Protein group	% Acidic residues	Conserved domain/ Protein family
RCC1731 G. huxleyi		
62	22.00%	14-3-3 protein
58	17.50%	Various DNA-binding
79	16.85%	Chromosome segregation ATPase Smc
49	14.00%	None
50	13.50%	Actin family
RCC1314*G. oceanica*		
110	21.15%	None
112	16.15%	14-3-3 protein
59	14.80%	Cupredoxin family
169	14.50%	Heat shock protein 90 family
92	14.20%	SAM (Sterile alpha motif)
RCC1198*C. braarudii*		
112	17.67%	14-3-3 protein
164	16.00%	None
144	15.90%	Chromosome segregation ATPase Smc
173	15.20%	None
197	15.00%	RNA recognition motif (RRM) superfamily

### The pentapeptide repeat appears a common feature of coccolith-associated proteins

Predominantly found in cyanobacteria, pentapeptide repeat-containing proteins are ubiquitously found across all lifeforms and are characterised by presence of repetitive [S, T,A, V][D, N][L, F][S, T,R][G] motifs, being fully comprised of, or containing domains composed of, this repetitive structure^83,84^. The precise function of these proteins remains unclear, but they are predicted to exist across all cellular compartments in cyanobacteria ranging from the cytoplasm to thylakoid and cell membrane^85^. Pentapeptide-repeat containing proteins were observed in each of the examined species. In total 36 (28%) of the protein groups contained pentapeptide-repeat containing proteins, with the majority (30) being identified in the *G. oceanica* dataset. In previous works conducted by Skeffington et al. (2023) on *G. huxleyi* this structural motif has also been highlighted as a feature of *G. huxleyi* coccolith-associated proteins. Despite limited knowledge on the functionality of pentapeptide repeat-containing proteins in general, several have been investigated in greater detail. One such example is the Heterocyst Glycolipid Biosynthesis HglK protein of *Nostoc sp.* strain PCC 7120. This protein is believed to be necessary for glycolipid localisation on heterocyst walls^85^. The presence of the pentapeptide repeat in previous work on *G. huxleyi* and across all species examined in our work, suggests a conservation of this structural feature within the coccolith matrix and offers scope for targeted research on the role of these proteins in coccolithogenesis.

Owing to the apparent conserved nature of pentapeptide-repeat containing proteins in the coccolith matrix, all identified proteins obtained for each species were submitted for sequence alignment using the NCBI BLASTp tool^57^. Results were filtered to focus on those proteins displaying a high degree of similarity (> 90%). While neither of the two pentapeptide-repeat proteins identified in *C. braarudii* were found to display high similarity to those identified in *G. huxleyi* or *G. oceanica*, all but one protein across each of the pentapeptide-repeat containing protein groups in *G. huxleyi* was found to have a > 90% identity match to those derived from *G. oceanica*. This included the two pentapeptide-repeat proteins proposed as coccolith proteins by Skeffington et al. (2023) and predicted to be specific to the Gephyrocapsa genus^45^. Notably, three of the four other overlapping proteins between ours and the Skeffington et al. (2023) dataset, each predicted to be coccolithophore-specific and containing no conserved domain but a signal peptide region, displayed a 95–97% identity match to proteins observed in *G. oceanica*. This consistency provides greater support to their proposal as potential coccolith proteins in this genus^45^.

Two of the pentapeptide repeat-containing proteins identified in *G. oceanica* contained a further domain of interest. This modular organisation, inferring multifunctionality, is a property reported in proteins associated with the shell matrix of bivalves^86^. First, CAE5880018.1 (protein group 36) additionally contained a PAN_APPLE domain region found in plant receptor-like protein kinases and which fulfil a range of biological functions involved with protein-carbohydrate or protein-protein interactions^87^. This protein was predicted by InterProScan to contain a signal peptide and was characterised as non-cytoplasmic, suggesting its possible targeting and localisation in the CV. An additional protein (protein group 25; CAE5907365.1) containing the PAN_APPLE domain was also identified in *G. oceanica.* Second, the pentapeptide-repeat protein, CAG0045004.1 (protein group 120), contained a match to the Core-2 branching enzyme domain in CD-Search and was predicted to belong to the glycosyl transferase 14 family by InterProScan. Core-2 branching enzyme is a glycosyltransferase protein known to play a key role in O-glycan side-chain branch formation and typically situated in the ER or Golgi^88,89^. Such a finding is of interest given the known function of CAPs in controlling calcite crystal nucleation and growth^28^. Little additional functional information could be gained from *G. huxleyi* and *C. braarudii* pentapeptide-repeat proteins.

The association of repetitive protein structures in the process of biomineralisation appears widespread^35^. Such structures have been identified in a number of marine calcifying organisms including bivalves and echinoderms^33–37^. The pentapeptide repeat, specifically, has previously been characterised in the highly-acidic aspein protein of pearl oysters, believed to direct calcite formation^65^. Given this consistency, it is feasible to suggest that pentapeptide repeats may play a role in controlling calcite formation during coccolithogenesis. Further work is required to demonstrate the role of repeating motifs in calcification by coccolithophores, whereby synthetic methods will likely provide critical insight^35^.

### Cell signalling & protein modification

Given the belief that calcification is under strict biological control^1^, its regulation must involve a number of cell signalling processes. Indeed, cell signalling proteins have been identified in the shell/skeletal matrix of mollusc and coral species^36,37,86^. Herein, 14-3-3 family proteins were identified in all examined species. This acidic protein domain is typically involved in signal transduction and protein targeting^82^. In Arabidopsis 14-3-3- proteins bind Ca^2+^ and act as a molecular switch to mediate protein kinase activity and trigger the response to stress^90^. The presence of 14-3-3 proteins in the coccolith matrix may suggest a possible role in a calcification signalling pathway, although further work is required to confirm this. Notably, BLASTp alignment revealed a high degree of similarity between the 14-3-3 proteins identified in *G. huxleyi* and *G. oceanica* (up to 99.6%; 3.06E-174), suggestive of a possible conserved function.

A number of additional protein modification/cell signalling related domains were identified in *G. oceanica*. Protein group 92 was observed to contain two Sterile alpha motif (SAM) domains which function as a protein interaction module for a wide variety of proteins across a number of cell processes^91^. The presence of a signal peptide on this protein suggests it may be localised to the CV. Additionally, a leucine-rich repeat-containing (LRR) plant receptor-like serine/threonine kinase (protein group 134; CAE5870709.1) was recorded in *G. oceanica*. Typically, the LRR is involved in protein-protein interaction^92^, and LRR proteins operate in a wide variety of cellular processes including signal transduction, cell adhesion, DNA repair and transcriptional regulation. In plants, the LRR receptor-like protein kinase domain has been implicated in the control of plant development and growth^93^. Previously, the LRR has also been recorded in proteins derived from the coccoliths of *G. huxleyi*^45^. Ubiquitin-like domains which act in protein ubiquitination were also found in two protein groups (109 and 135) in *G. oceanica*, as well as one (108) in *C. braarudii*. Ubiquitin-related domains have been previously reported in the skeletal matrix of the stony coral *S. pistilla*^36^. Several methyltransferase domain- containing protein groups were identified in *G. oceanica*, two of which contained signal peptide regions.

Post-translational modifications (PTMs) mediate activity for the majority of eukaryotic proteins, dictating their turnover, localisation, and interaction with other cellular components^94^. PEAKs PTM search predicted the majority of proteins identified to contain at least one PTM (Supplementary Data Table 1). Further work is required to accurately assess whether such modifications are true PTMs, or if their occurrence is related to the sample preparation methods utilised. For example, oxidation of methionine residues by H_2_O_2_ is well characterised^95^. It is possible that post-translational modification could mediate the role of specific proteins present within the CV and growing coccolith during coccolithogenesis. For example, presence of sulfonated groups has been reported to inhibit calcification on surface biomaterials due to their negative charge inducing rapid formation of Ca-P compounds and localised reduction in pH increasing the solubility of Ca^2+^ and hence preventing crystal nucleation^96–99^. Future research confirming the presence and possible role of PTMs in coccolithogenesis would be highly beneficial.

### Protein folding, proteolysis & protease inhibition appear associated with calcite structures

In both *G. oceanica* and *C. braarudii* coccoliths protein groups with conserved domains relating to protein folding, degradation and protection were identified. Members of the peptidylprolyl isomerase (PPIase) family function in protein folding^100^. Two subgroups of the PPIase family were identified in *G. oceanica*, cyclophilin (protein group 111) and the FKBP-type PPIase (protein group 138), both of which were predicted to contain a signal peptide region. Cyclophilins possess typical PPIase function, but also specific domains pertaining to precise selection of protein substrate or localisation within the cell^101^. Cyclophilin proteins have previously been identified in the calcified structures of other marine calcifying organisms including crayfish and both adult and sea urchin larvae^102–104^.

Proteins with functions relating to proteolysis i.e., protein degradation, were observed in *G. oceanica* and *C. braarudii* coccoliths. In *G. oceanica* this included four peptidase protein groups, three of which contained a signal peptide. Here, members of the C1 (protein group 43) and C13 (protein group 93) peptidase families were identified, as well as one member of the subtilisin-like serine protease family (protein group 26) and glutamyl endopeptidase family (protein group 104), respectively. Peptidase-containing proteins have also been reported in the skeletal matrix of corals^37^. Related to this, protease inhibitors have previously been identified in skeletal matrices, where they may provide protection from proteolytic activity^71,105,106^. A cystatin-like domain-containing protein (protein group 95) was observed in *G. oceanica* coccoliths. Cystatin belongs to the cysteine protease inhibitor family, but is also associated with a number of extracellular proteins^107^. This protein contained a signal region and was predicted to be non-cytoplasmic, possibly being localised to the CV. Inhibition of proteases by cystatins are reversible, allowing for potential control of protease activity. The major cysteine proteases which interact with cystatin include the papain family, derived from plants^107^. Interestingly, within the *G. oceanica* dataset we also identified a C1 peptidase papain family protein (protein group 43) which too contained a signal peptide region and was predicted to be non-cytoplasmic. It is possible that these two proteins interact with one another during coccolithogenesis in *G. oceanica*, however future work is required to confirm this. Another member of the cysteine protease inhibitor family Lustrin A, whose cysteine and proline-rich structure shows similarity to frustulins involved in silicification in diatoms^108,109^, is implicated in the calcification pathway of bivalve species. Lustrin A is proposed to protect the secreted proteins of the shell matrix from degradation^26^. A cysteine rich trypsin-like protease inhibitor has also been identified in the skeletal matrix of the stony coral *S. pistillata*^36^. It is feasible to suggest, given this consistency, that cysteine-rich protease inhibitors may play a broader role in the process of biomineralisation by protecting calcification proteins from degradation.

### A possible role for carbohydrate metabolism proteins in coccolithogenesis

Calcification and central metabolism must be interconnected in coccolithophores, given that photosynthetic energy must be directed towards calcification and products converted into the key metabolites such as CAPs, and structural components required for coccolithogenesis^29^. In addition to the Core-2 branching enzyme domain mentioned in the previous section, a further four protein groups were identified in *G. oceanica* with possible roles relating to carbohydrate metabolism. It is feasible to suggest that proteins involved in the modification of carbohydrate structures could be involved in coccolithogenesis, given the critical role of CAPs in controlling crystal growth^28^.

A glycosyltransferase family protein domain was identified in *G. oceanica* (protein group 38; CAG0097667.1). Presence of the glycosyltransferase domain has also been identified in the coccolith matrix of *G. huxleyi* by Skeffington et al.. (2023). Glycotransferases function in polysaccharide and oligosaccharide synthesis, or production of glycoconjugates by transfer of a sugar group^110^. Following functional analysis, CAG0097667.1 was predicted to additionally contain a CUB domain. A number of proteins containing the CUB domain have been identified in the skeletal organic matrix of the stony coral *Acropora millepora*^37^. This typically extracellular domain was first identified in calcium-dependent blood coagulation proteins Cls and Clr, embryonic sea urchin protein Uegf and bone morphogenetic protein (Bmp1)^111^. Bmp1 plays a diversity of roles including cartilage and bone formation, as well as collagen dynamics, and is implicated in pattern formation during development of a variety of organisms^112^. The presence of a protein sequence containing both a saccharide-modification domain and a functional domain, known to play a role in biomineralisation, is intriguing in terms of coccolithogenesis.

Three protein groups identified in *G. oceanica* were observed to contain glycosyl/glycoside hydrolyse domains (protein groups 114; 130; 139), two of which contained signal peptide regions, possibly locating them to the CV. Such proteins act to hydrolase carbohydrate bonds, contributing to their degradation^113^. For example, protein group 114 (CAE5907845.1) possessed a signal peptide region and was annotated as alpha-L-arabinofuranosidase. This enzyme is typically involved in degradation of arabinoxylan which represents a large proportion of plant hemicellulose^114^. Coccoliths form around an organic baseplate, which is believed to act as a scaffold to direct and control crystal nucleation and growth and is a constituent of each coccolith^115^. This baseplate is proposed to be composed of layers of a fibrous, in some cases, cellulose-like texture^44,116^. The presence of carbohydrate degradation proteins within the CV, including those relating to cellulosic-like molecule degradation, could act to regulate the influence of the baseplate and CAPs on coccolith formation, or to recycle these materials and for future rounds of coccolithogenesis. Here, metabolomic analyses may aid our understanding of CAP metabolism during and after coccolith production.

### The cytoskeleton & basic cellular processes

Despite a clear role of cytoskeletal proteins in coccolithogenesis^25^, little additional information regarding their function, or presence within coccoliths was gained in our study, despite being recorded in previous work^45^. It is possible the lack of identification may also arise due to similarities in highly conserved cytoskeletal proteins which are included as potential contaminants during protein database searches. Actin, a core component of the cytoskeleton, was identified in the coccoliths of *G. huxleyi* only (protein group 50). Actin has previously been recorded during analysis of proto-coccolith fractions of *G. huxleyi*^45^. The microtubular protein, kinesin, was identified in *C. braarudii* (protein group 172), whilst a fibronectin domain was recorded in *G. oceanica* (protein group 100). Fibronectin is a high molecular weight glycoprotein which operates in a number of cell functions, including cell adhesion^117^. These domains have previously been observed to be present in the skeletal matrix of coral species^36,37^. Similarly, the MAM domain which also has functions relating to cell adhesion was previously identified in the coccoliths of *G. huxleyi*^45,118^. Finally, presence of a plant cell wall pistil-specific extensin-like protein (protein group 170; CAE5912150.1) was found in *G. oceanica*. Extensin proteins provide strengthening to the plant cell wall and are characterised by multiple pentapeptide-repeat sequences^119^. Intriguingly, a candidate CV protein described by Skeffington et al. (2023) (EhG42278.1) also displayed characteristic features of the extensin family and is proposed to contribute to baseplate assembly^45^. While, no significant similarity was present between the protein identified in our work and EhG42278.1, CAE5912150.1 could play a similar role or contribute to overall coccolith structural integrity in *G. oceanica*. Indeed, further research of extension-like proteins in coccolithophores would be beneficial.

We recorded a number of proteins with functions related to core cellular processes such as helicases, observed in *C. braarudii* only, as well as ribosomal proteins and chromosome segregation ATPase Smc-domain containing proteins, of which the latter was recorded in both *G. huxleyi* (protein group 79) and *C. braarudii* (protein group 144). Such proteins do not have an obvious role in calcification and may represent cellular contamination, however, these proteins were not identified in the control. Structural maintenance of chromosomes (SMC) proteins are widely conserved, acting to manipulate genome structure through intra- and intermolecular protein-protein interaction^120^. Presence of this domain may represent cellular contamination, however, a protein containing a significant match for alpha-tubulin suppressor and related RCC1 domain-containing proteins was recorded during CD-Search of the proposed coccolith proteins in previous work (EhG32342.1)^45^. This domain also typically functions in chromosome partitioning and cytoskeletal dynamics but has additionally been found to display a wider range of functions including interaction with protein and lipid molecules^121^. Of note, the RCC1 domain typically interacts with the nuclear GTPase Ran protein^122^. Presence of a Ran GTPase domain containing protein was recorded in *G. oceanica* (protein group 84) in our work. The typical function of Ran is in nucleocytoplasmic protein transport^123^, however the small GTPase family to which Ran belongs, includes some which contribute to vesicular trafficking pathways and cytoskeletal dynamics^124^, both of which are relevant to coccolithogenesis.

Several histone-related proteins were identified during analysis in both the control and coccolith samples derived from *G. huxleyi* (protein group 57) and *C. braarudii* (protein groups 92 and 97). Histones function in chromatin organisation, making up a major component of the nucleosome^125^. In species such as *G. huxleyi* and *C. braarudii*, the CV is located in direct contact with the nucleus^15^, this localisation may increase the likelihood of histone proteins binding to the coccolith and explain their presence in our dataset.

## Conclusions

**Fig. 4:**
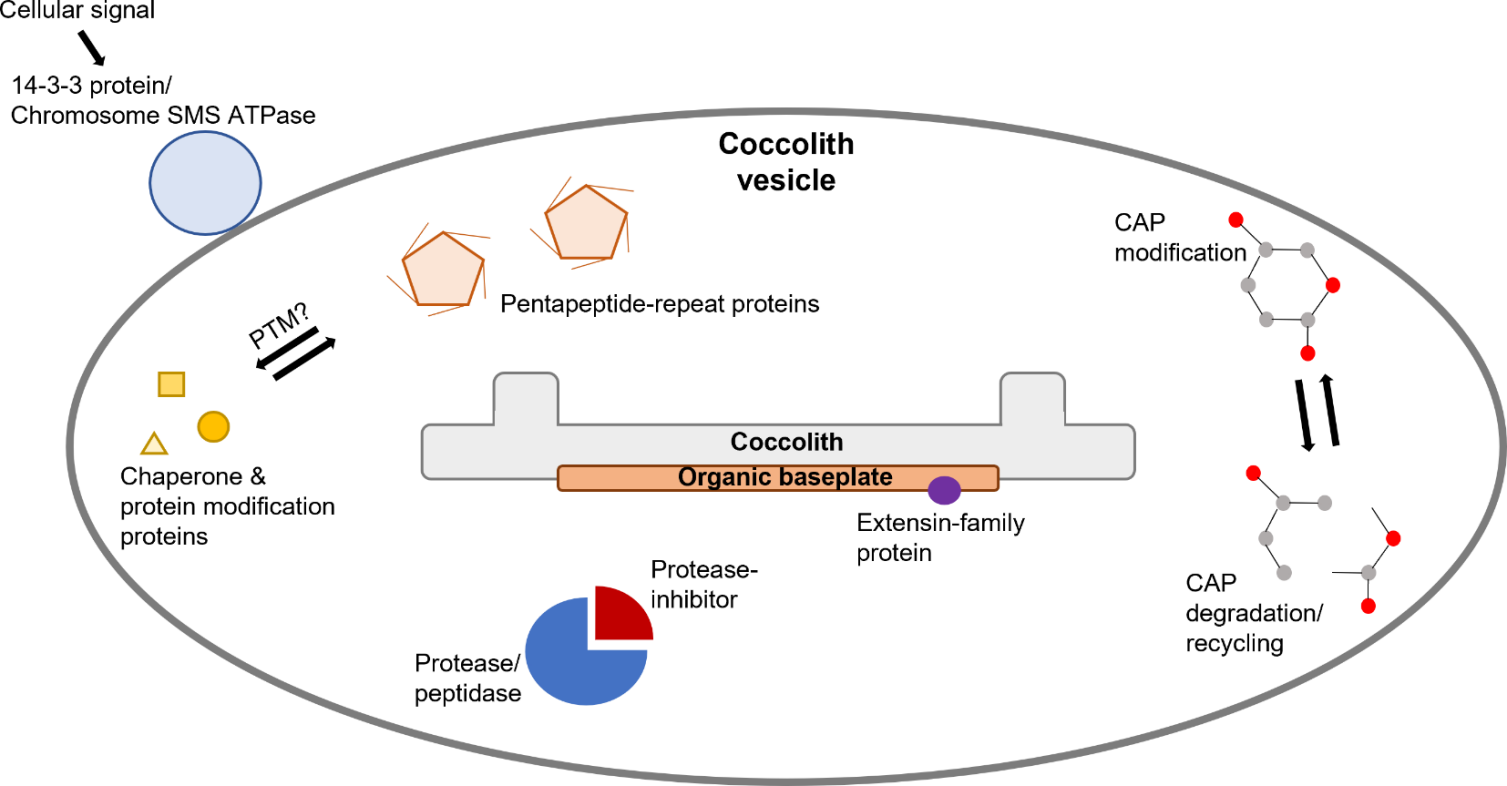
Graphical representation of common protein features and/or inferred functions identified across the coccolith proteins identified in this study.

Coccolithophores play a critical role in the marine carbon cycle, however the mechanisms which regulate and control their calcification activity remain unresolved. In this work, we have explored the presence and possible function of proteins incorporated within the coccolith scales of coccolithophores, building on the pioneering work of Skeffington et al. (2023). For the first time, we present potential coccolith proteins derived from *G. oceanica* and *C. braarudii*, representing key models of coccolithophore research. Cross-referencing between our work, that conducted by Skeffington et al. (2023) and previous studies on marine calcifying organisms, we identify specific proteins and functional features of interest for future research (Fig. 4), as well as commonalities across biomineralisers. Notably, in-line with existing findings on coccolith proteins, and in accordance with proteins identified in the calcite matrix of other marine calcifying organisms, we suggest that repetitive motifs likely play an important role in crystal formation and growth. Specifically, for coccolithophores the pentapeptide repeat appears to be a structural feature associated with the coccolith matrix, offering scope for targeted research to confirm their likely role.

We identify that protein domains involved in cell signalling and protein-protein interaction are present within coccoliths, consistent with those proteins identified in calcium carbonate structures produced by other marine calcifiers. The highly acidic 14-3-3 domain is of interest given its occurrence in each of the three examined species, whereby its potential to bind and respond to Ca^2+^ is a feature of particular interest. We suggest that post-translational modification could play a part in determining the function of CV and coccolith proteins by altering physicochemical properties, i.e., electrostatic charge and hydrophobicity, which could influence interaction with CAPs or Ca^2+^. In previous work, GPA (glutamine, proline, alanine-rich protein) has been proposed to play a role in coccolithophore calcification, however its function and expression profile is not clear^126,127^. We did not identify GPA in our work, nor was this protein identified in previous analyses of *G. huxleyi* coccoliths or coccosphere-associated proteins, suggesting this protein may not directly interact with the growing coccolith^45^. Notably, we did not identify any protein with a defined Ca^2+^-binding domain in any species, despite Ca^2+^-binding proteins being identified in other marine calcifiers^36,37,128–130^. Though, the copper-binding cupredoxin domain, present in the coral skeletal matrix hephaestin-like protein, was identified in both *Gephyrocapsa* species examined, possibly, suggestive of a role of copper in biomineralisation. A feature shared by a variety of marine calcifying organisms, including coccolithophores, is the presence of protease inhibitors within the calcite/shell matrix, which may play a critical role in ensuring effective calcification activity and morphology by protecting those proteins involved in directing crystal growth from degradation. A key finding in our work is the consistent identification of several *G. huxleyi* coccolith proteins, also identified by Skeffington et al. (2023). These proteins listed in Table SI.5 represent strong candidates for future work on deciphering the molecular control of calcification in this core model of coccolithophore biology.

Additional research is required to fully capture the repertoire of proteins which are incorporated into the coccoliths of coccolithophores. Identification of these proteins will undoubtedly enhance our understanding of the likely calcification pathway in these organisms. A key point remains that in our study (50 L) and in previous work (100 L)^45^, large culture volumes were required to ensure sufficient coccolith material for proteomic analysis, indicating that the protein content of coccoliths is low. Hence, debate exists over how influential a role this relatively small component could hold on overall coccolith formation. Additional research is required to address this question. The works presented offer a number of targets for future research, where synthetic methods may be key to fully establishing the role of proteins in coccolithogenesis. New genomic information may allow for interrogation of variation in protein sequence and/or structure between species and strains which may reveal the mechanistic controls which confer the vast differences observed in coccolith morphology. Here, production of reference genomic databases for additional coccolithophore species will be invaluable. Similarly, developments in our limited ability to manipulate the genome of coccolithophore models^16,131^, will facilitate the generation of mutant strains allowing gene knock-outs for proposed coccolith proteins, resulting in more detailed assessment of their role in this process. Importantly, work is required to understand the interplay between coccolith proteins and CAPs, and the specific biochemical composition of the baseplate and growing crystal surface upon which they interact.

## Electronic Supplementary Material

Below is the link to the electronic supplementary material.

Supplementary Material 1

Supplementary Material 2

Supplementary Material 3

## Data Availability

The mass spectrometry proteomics data have been deposited to the ProteomeXchange Consortium (http://proteomecentral.proteomexchange.org) via the PRIDE partner repository with the dataset identifier PXD054749^132^.

## References

[1] Paasche, E. A review of the coccolithophorid Emiliania huxleyi (Prymnesiophyceae), with particular reference to growth, coccolith formation, and calcification-photosynthesis interactions. *Phycologia* **40** (6), 506–529 (2001).

[2] Broecker, W. & Clark, E. Ratio of coccolith CaCO_3_ to foraminifera CaCO_3_ in late Holocene deep sea sediments. *Paleoceanography* **24** (3). 10.1029/2009PA001731 (2009).

[3] Schiebel, R. Planktic foraminiferal sedimentation and the marine calcite budget. *Glob. Biogeochem. Cycles*. **16** (4), 3–1. 10.1029/2001GB001459 (2002).

[4] Krumhardt, K. M., Lovenduski, N. S., Iglesias-Rodriguez, M. D. & Kleypas, J. A. Coccolithophore growth and calcification in a changing ocean. *Progress Oceanogr.* **159**, 276–295 (2017).

[5] Stoll, H. M. & Ziveri, P. Coccolithophorid-based geochemical paleoproxies. In Coccolithophores: From Molecular Processes to Global Impact, (eds Thierstein, H. R. & Young, J. R.) Eds.; Springer Berlin Heidelberg, ; 529–562. (2004).

[6] Stoll, H. M., Rosenthal, Y. & Falkowski, P. Climate proxies from Sr/Ca of coccolith calcite: Calibrations from continuous culture of Emiliania huxleyi. *Geochim. Cosmochim. Acta*. **66** (6), 927–936. 10.1016/S0016-7037(01)00836-5 (2002).

[7] Claxton, L. M., McClelland, H. L. O., Hermoso, M. & Rickaby, R. E. M. Eocene emergence of highly calcifying coccolithophores despite declining atmospheric CO2. *Nat. Geosci.* **15** (10), 826–831. 10.1038/s41561-022-01006-0 (2022).

[8] Rickaby, R. E. M. et al. Coccolith chemistry reveals secular variations in the global ocean carbon cycle? *Earth Planet. Sci. Lett.* **253** (1), 83–95. 10.1016/j.epsl.2006.10.016 (2007).

[9] Bendif, E. M. et al. Repeated species radiations in the recent evolution of the key marine phytoplankton lineage Gephyrocapsa. *Nat. Commun.* **10** (1), 4234. 10.1038/s41467-019-12169-7 (2019). 31530807 10.1038/s41467-019-12169-7 PMC6748936

[10] Bendif, E. M. et al. Rapid diversification underlying the global dominance of a cosmopolitan phytoplankton. *ISME J.* **17** (4), 630–640. 10.1038/s41396-023-01365-5 (2023). 36747097 10.1038/s41396-023-01365-5 PMC10030636

[11] Brownlee, C., Langer, G. & Wheeler, G. L. Coccolithophore calcification: Changing paradigms in changing oceans. *Acta Biomater.* **120**, 4–11. 10.1016/j.actbio.2020.07.050 (2021). 32763469 10.1016/j.actbio.2020.07.050

[12] Wheeler, G. L., Sturm, D. & Langer, G. Gephyrocapsa huxleyi (Emiliania huxleyi) as a model system for coccolithophore biology. *J. Phycol.* **59** (6), 1123–1129. 10.1111/jpy.13404 (2023). 37983837 10.1111/jpy.13404

[13] van der Wal, P., de Jong, E. W., Westbroek, P., de Bruijn, W. C. & Mulder-Stapel, A. A. Polysaccharide localization, coccolith formation, and golgi dynamics in the coccolithophorid Hymenomonas carterae. *J. Ultrastruct. Res.* **85** (2), 139–158. 10.1016/S0022-5320(83)90103-X (1983). 6425510 10.1016/s0022-5320(83)90103-x

[14] Westbroek, P. et al. Mechanism of calcification in the marine alga Emiliania huxleyi. *Phil Trans. R Soc. B*. **304**, 435–444 (1984).

[15] Taylor, A. R., Russell, M. A., Harper, G. M., Collins, T. T. & Brownlee, C. Dynamics of formation and secretion of heterococcoliths by Coccolithus pelagicus ssp. braarudii. *Eur. J. Phycol.* **42**(2), 125–136. 10.1080/09670260601159346 (2007).

[16] Taylor, A. R., Brownlee, C. & Wheeler, G. Coccolithophore cell biology: Chalking up progress. *Ann. Rev. Mar. Sci.* **9**, 283–310. 10.1146/annurev-marine-122414-034032 (2017). 27814031 10.1146/annurev-marine-122414-034032

[17] Brownlee, C. & Taylor, A. Calcification in coccolithophores: A cellular perspective. In Coccolthophores, (eds Thierstein, H. R. & Young, J. R.) (Springer, 2004).

[18] Anning, T., Nimer, N., Merrett, M. J. & Brownlee, C. Costs and benefits of calcification in coccolithophorids. *J. Mar. Syst.* **9** (1), 45–56. 10.1016/0924-7963(96)00015-2 (1996).

[19] Klaveness, D. Morphologic investigations on the vegetative cell and the process of coccolith formation. *Protistological* **8**, 335–346 (1972).

[20] Didymus, J. M., Young, J. R. & Mann, S. Construction and morphogenesis of the chiral ultrastructure of coccoliths from the marine alga Emiliania huxleyi. *Proc. R Soc. Lond. B*. **258**, 237–245 (1994).

[21] Marsh, M. E. Polyanion-mediated mineralization - assembly and reorganization of acidic polysaccharides in the golgi system of a coccolithophorid alga during mineral deposition. *Protoplasma* **177**, 108–122 (1994).

[22] Young, J. R., Davis, S. A., Bown, P. R. & Mann, S. Coccolith ultrastructure and biomineralisation. *J. Struct. Biol.* **126** (3), 195–215. 10.1006/jsbi.1999.4132 (1999). 10441529 10.1006/jsbi.1999.4132

[23] Marsh, M. E., Ridall, A. L., Azadi, P. & Duke, P. J. Galacturonomannan and Golgi-derived membrane linked to growth and shaping of biogenic calcite. *J. Struct. Biol.* **139** (1), 39–45. 10.1016/s1047-8477(02)00503-8 (2002). 12372318 10.1016/s1047-8477(02)00503-8

[24] Young, J. R., Andruleit, H. & Probert, I. Coccolith function and morphogenesis: Insights from appendage-bearing coccolithophores of the family syracosphaeraceae (Haptophyta)(1). *J. Phycol.* **45** (1), 213–226. 10.1111/j.1529-8817.2008.00643.x (2009). 27033659 10.1111/j.1529-8817.2008.00643.x

[25] Langer, G. et al. The effect of cytoskeleton inhibitors on coccolith morphology in Coccolithus braarudiiand Scyphosphaera apsteinii. *J. Phycol.* **59** (1), 87–96 (2022). 36380706 10.1111/jpy.13303

[26] Zhang, C. & Zhang, R. Matrix proteins in the outer shells of molluscs. *Mar. Biotechnol.* **8** (6), 572–586. 10.1007/s10126-005-6029-6 (2006). 10.1007/s10126-005-6029-6 16614870

[27] Walker, J. M. & Langer, G. Coccolith crystals: Pure calcite or organic-mineral composite structures? *Acta Biomater.* **125**, 83–89. 10.1016/j.actbio.2021.02.025 (2021). 33631395 10.1016/j.actbio.2021.02.025

[28] Henriksen, K., Stipp, S. L. S., Young, J. R. & Marsh, M. E. Biological control on calcite crystallization: AFM investigation of coccolith polysaccharide function. *Am. Mineral.* **89** (11–12), 1709–1716. 10.2138/am-2004-11-1217 (2004).

[29] Monteiro, F. M. et al. Why marine phytoplankton calcify. *Sci. Adv. 2* (7), e1501822 10.1126/sciadv.1501822. (2016). 10.1126/sciadv.1501822 PMC4956192 27453937

[30] Fichtinger-Schepman, A. J., Kamerling, J. P., Versluis, C. & Vliegenthart, J. F. G. Structural studies of the methylated, acidic polysaccharide associated with coccoliths of Emiliania huxleyi (Lohmann) Kamptner. *Carbohydr. Res.* **93**, 105–123 (1981).

[31] Ozaki, N., Sakuda, S. & Nagasawa, H. A novel highly acidic polysaccharide with inhibitory activity on calcification from the calcified scale coccolith of a coccolithophorid alga, Pleurochrysis haptonemofera. *Biochem. Biophys. Res. Commun.* **357** (4), 1172–1176. 10.1016/j.bbrc.2007.04.078 (2007). 17462599 10.1016/j.bbrc.2007.04.078

[32] Weiner, S. & Hood, L. Soluble protein of the organic matrix of mollusk shells: A potential template for shell formation. *Science* **190** (4218), 987–989. 10.1126/science.1188379 (1975). 1188379 10.1126/science.1188379

[33] Wheeler, A. P., George, J. W. & Evans, C. A. Control of calcium carbonate nucleation and crystal growth by soluble matrx of oyster shell. *Science* **212** (4501), 1397–1398. 10.1126/science.212.4501.1397 (1981). 17746262 10.1126/science.212.4501.1397

[34] Du, C., Falini, G., Fermani, S., Abbott, C. & Moradian-Oldak, J. Supramolecular assembly of amelogenin nanospheres into birefringent microribbons. *Science* **307** (5714), 1450–1454. 10.1126/science.1105675 (2005). 15746422 10.1126/science.1105675

[35] Shiba, K. & Minamisawa, T. A. Synthesis approach to understanding repeated peptides conserved in mineralization proteins. *Biomacromolecules* **8** (9), 2659–2664. 10.1021/bm700652b (2007). 17665949 10.1021/bm700652b

[36] Drake, J. L. et al. Proteomic analysis of skeletal organic matrix from the stony coral Stylophora pistillata. *Proceedings of the National Academy of Sciences 110* (10), 3788–3793. DOI: (2013). 10.1073/pnas.1301419110 10.1073/pnas.1301419110 PMC3593878 23431140

[37] Ramos-Silva, P. et al. The skeletal proteome of the coral Acropora millepora: The evolution of calcification by co-option and domain shuffling. *Mol. Biol. Evol.* **30** (9), 2099–2112. 10.1093/molbev/mst109 (2013). 23765379 10.1093/molbev/mst109 PMC3748352

[38] Song, X., Liu, Z., Wang, L. & Song, L. Recent advances of shell matrix proteins and cellular orchestration in marine molluscan shell biomineralization. *Front. Mar. Sci.* **6**10.3389/fmars.2019.00041 (2019).

[39] Mummadisetti, M. P., Drake, J. L. & Falkowski, P. G. The spatial network of skeletal proteins in a stony coral. *J. R Soc. Interface*. **18** (175), 20200859. 10.1098/rsif.2020.0859 (2021). 33622149 10.1098/rsif.2020.0859 PMC8086859

[40] Jiang, W. et al. D. Chiral acidic amino acids induce chiral hierarchical structure in calcium carbonate. *Nat. Commun.* **8** (1), 15066. 10.1038/ncomms15066 (2017). 28406143 10.1038/ncomms15066 PMC5399303

[41] Meierhenrich, U. J. Amino acids and the asymmetry of life. *Eur. Rev.* **21** (2), 190–199. 10.1017/S106279871200035X (2013). From Cambridge University Press Cambridge Core.

[42] Lee, R. B. Y., Mavridou, D. A. I., Papadakos, G., McClelland, H. L. O. & Rickaby, R. E. M. The uronic acid content of coccolith-associated polysaccharides provides insight into coccolithogenesis and past climate. *Nat. Commun.* **7** (1), 13144. 10.1038/ncomms13144 (2016). 27782214 10.1038/ncomms13144 PMC5095175

[43] Hood, M. A., Leemreize, H., Scheffel, A. & Faivre, D. Lattice distortions in coccolith calcite crystals originate from occlusion of biomacromolecules. *J. Struct. Biol.* **196** (2), 147–154. 10.1016/j.jsb.2016.09.010 (2016). 27645701 10.1016/j.jsb.2016.09.010

[44] Walker, J. M. et al. Polymorph selectivity of coccolith-associated polysaccharides from gephyrocapsa oceanica on calcium carbonate formation in vitro. *Adv. Funct. Mater.* **29** (1), 1807168. 10.1002/adfm.201807168 (2019).

[45] Skeffington, A. et al. A joint proteomic and genomic investigation provides insights into the mechanism of calcification in coccolithophores. *Nat. Commun.* **14** (1), 3749. 10.1038/s41467-023-39336-1 (2023). 37353496 10.1038/s41467-023-39336-1 PMC10290126

[46] Liu, H., Aris-Brosou, S., Probert, I. & de Vargas, C. A time line of the environmental genetics of the haptophytes. *Mol. Biol. Evol.* **27** (1), 161–176. 10.1093/molbev/msp222 (2010). 19762334 10.1093/molbev/msp222

[47] Price, N. M. et al. Preparation and chemistry of the artificial algal culture medium aquil. *Biol. Oceanogr.* **6** (5–6), 443–461. 10.1080/01965581.1988.10749544 (1989).

[48] Keller, M. D., Selvin, R. C., Claus, W. & Guillard, R. R. L. Media for the culture of oceanic ultraphytoplankton 1,2. *J. Phycol.* **23** (4), 633–638. 10.1111/j.1529-8817.1987.tb04217.x (1987).

[49] Rickaby, R. E. M., Henderiks, J. & Young, J. N. Perturbing phytoplankton: Response and isotopic fractionation with changing carbonate chemistry in two coccolithophore species. *Clim. Past*. **6** (6), 771–785. 10.5194/cp-6-771-2010 (2010).

[50] Christie-Oleza, J. A. & Armengaud, J. In-depth analysis of exoproteomes from marine bacteria by shotgun liquid chromatography-tandem mass spectrometry: The Ruegeria pomeroyi DSS-3 case-study. *Mar. Drugs*. **8** (8), 2223–2239. 10.3390/md8082223 (2010). 20948905 10.3390/md8082223 PMC2953401

[51] Saveliev, S. et al. Trypsin/Lys-C protease mix for enhanced protein mass spectrometry analysis. *Nat. Methods*. **10** (11), i–ii. 10.1038/nmeth.f.371 (2013).

[52] Marchler-Bauer, A. et al. CDD: NCBI’s conserved domain database. *Nucleic Acids Res.* **43** (D1), D222–D226. 10.1093/nar/gku1221 (2015). 25414356 10.1093/nar/gku1221 PMC4383992

[53] Gasteiger, E. et al. Protein identification and analysis tools on the ExPASy server. In *The Proteomics Protocols Handbook*, (ed Walker, J. M.) 571–607 (Humana Press, 2005).

[54] Zhang, J. et al. PEAKS DB: De Novo sequencing assisted database search for sensitive and accurate peptide identification. *Mol. Cell. Proteom.* **11** (4). 10.1074/mcp.M111.010587 (2012). 10.1074/mcp.M111.010587 PMC3322562 22186715

[55] Blum, M. et al. The InterPro protein families and domains database: 20 years on. *Nucleic Acids Res.* **49** (D1), D344–D354. 10.1093/nar/gkaa977 (2021). 33156333 10.1093/nar/gkaa977 PMC7778928

[56] Jones, P. et al. InterProScan 5: Genome-scale protein function classification. *Bioinformatics* **30** (9), 1236–1240. 10.1093/bioinformatics/btu031 (2014). 24451626 10.1093/bioinformatics/btu031 PMC3998142

[57] Camacho, C. et al. BLAST+: Architecture and applications. *BMC Bioinform.* **10**, 421. 10.1186/1471-2105-10-421 (2009). 10.1186/1471-2105-10-421 PMC2803857 20003500

[58] Yano, M., Nagai, K., Morimoto, K. & Miyamoto, H. Shematrin: A family of glycine-rich structural proteins in the shell of the pearl oyster Pinctada fucata. *Comp. Biochem. Physiol. B Biochem. Mol. Biol.* **144** (2), 254–262. 10.1016/j.cbpb.2006.03.004 (2006). 16626988 10.1016/j.cbpb.2006.03.004

[59] McDougall, C., Aguilera, F. & Degnan, B. M. Rapid evolution of pearl oyster shell matrix proteins with repetitive, low-complexity domains. *J. R. Soc. Interface*. **10** (82), 20130041. 10.1098/rsif.2013.0041 (2013). 23427100 10.1098/rsif.2013.0041 PMC3627088

[60] Michenfelder, M. et al. Characterization of two molluscan crystal-modulating biomineralization proteins and identification of putative mineral binding domains. *Biopolymers* **70** (4), 522–533. 10.1002/bip.10536 (2003). 14648763 10.1002/bip.10536

[61] Sarashina, I. & Endo, K. The complete primary structure of molluscan shell protein 1 (MSP-1), an Acidic glycoprotein in the shell matrix of the scallop Patinopecten yessoensis. *Mar. Biotechnol.* **3** (4), 362–369. 10.1007/s10126-001-0013-6 (2001). 10.1007/s10126-001-0013-6 14961352

[62] Samata, T. et al. A new matrix protein family related to the nacreous layer formation of Pinctada fucata. *FEBS Lett.* **462** (1–2), 225–229. 10.1016/s0014-5793(99)01387-3 (1999). 10580124 10.1016/s0014-5793(99)01387-3

[63] Sudo, S. et al. Structures of mollusc shell framework proteins. *Nature* **387** (6633), 563–564. 10.1038/42391 (1997). 9177341 10.1038/42391

[64] Zhang, C., Xie, L., Huang, J., Liu, X. & Zhang, R. A novel matrix protein family participating in the prismatic layer framework formation of pearl oyster, Pinctada fucata. *Biochem. Biophys. Res. Commun.* **344** (3), 735–740. 10.1016/j.bbrc.2006.03.179 (2006). 16630535 10.1016/j.bbrc.2006.03.179

[65] Tsukamoto, D., Sarashina, I. & Endo, K. Structure and expression of an unusually acidic matrix protein of pearl oyster shells. *Biochem. Biophys. Res. Commun.* **320** (4), 1175–1180. 10.1016/j.bbrc.2004.06.072 (2004). 15249213 10.1016/j.bbrc.2004.06.072

[66] Suzuki, M. et al. An acidic matrix protein, Pif, is a key macromolecule for nacre formation. *Science* **325** (5946), 1388–1390. 10.1126/science.1173793 (2009). 19679771 10.1126/science.1173793

[67] Zhang, Y. et al. A novel matrix protein participating in the nacre framework formation of pearl oyster, Pinctada fucata. *Comp. Biochem. Physiol. B Biochem. Mol. Biol.* **135** (3), 565–573. 10.1016/s1096-4959(03)00138-6 (2003). 12831776 10.1016/s1096-4959(03)00138-6

[68] Yan, Y. et al. Novel Matrix Protein, PfY2, Functions as a Crucial Macromolecule during Shell Formation. *Sci. Rep.* **7** (1), 6021. 10.1038/s41598-017-06375-w (2017). 28729529 10.1038/s41598-017-06375-w PMC5519542

[69] Kono, M., Hayashi, N. & Samata, T. Molecular mechanism of the nacreous layer formation in Pinctada maxima. *Biochem. Biophys. Res. Commun.* **269** (1), 213–218. 10.1006/bbrc.2000.2274 (2000). 10694502 10.1006/bbrc.2000.2274

[70] Isowa, Y., Sarashina, I., Setiamarga, D. H. & Endo, K. A comparative study of the shell matrix protein aspein in pterioid bivalves. *J. Mol. Evol.* **75** (1–2), 11–18. 10.1007/s00239-012-9514-3 (2012). 22922907 10.1007/s00239-012-9514-3

[71] Conci, N., Lehmann, M., Vargas, S. & Wörheide, G. Comparative proteomics of octocoral and scleractinian skeletomes and the evolution of coral calcification. *Genome Biol. Evol.* **12** (9), 1623–1635. 10.1093/gbe/evaa162 (2020). 32761183 10.1093/gbe/evaa162 PMC7533068

[72] Luo, Y. J. et al. The Lingula genome provides insights into brachiopod evolution and the origin of phosphate biomineralization. *Nat. Commun.* **6** (1), 8301. 10.1038/ncomms9301 (2015). 26383154 10.1038/ncomms9301 PMC4595640

[73] Hilgers, L., Hartmann, S., Hofreiter, M. & von Rintelen, T. Novel genes ancient genes, and gene co-option contributed to the genetic basis of the Radula, a Molluscan innovation. *Mol. Biol. Evol.* **35** (7), 1638–1652. 10.1093/molbev/msy052 (2018). 29672732 10.1093/molbev/msy052 PMC5995198

[74] Fang, D. et al. Identification of genes directly involved in shell formation and their functions in pearl oyster, Pinctada fucata. *PLoS ONE*. **6** (7), e21860. 10.1371/journal.pone.0021860 (2011). 21747964 10.1371/journal.pone.0021860 PMC3128620

[75] Werner, G. D., Gemmell, P., Grosser, S., Hamer, R. & Shimeld, S. M. Analysis of a deep transcriptome from the mantle tissue of Patella vulgata Linnaeus (Mollusca: Gastropoda: Patellidae) reveals candidate biomineralising genes. *Mar. Biotechnol. (NY)*. **15** (2), 230–243. 10.1007/s10126-012-9481-0 (2013). 22865210 10.1007/s10126-012-9481-0

[76] Pan, C. et al. A novel acidic matrix protein, PfN44, stabilizes magnesium calcite to inhibit the crystallization of aragonite. *J. Biol. Chem.* **289** (5), 2776–2787. 10.1074/jbc.M113.504027 (2014). 24302723 10.1074/jbc.M113.504027 PMC3908410

[77] Suzuki, M., Iwashima, A., Kimura, M., Kogure, T. & Nagasawa, H. The molecular evolution of the pif family proteins in various species of mollusks. *Mar. Biotechnol. (NY)*. **15** (2), 145–158. 10.1007/s10126-012-9471-2 (2013). 22847736 10.1007/s10126-012-9471-2

[78] Shimizu, K., Negishi, L., Kurumizaka, H. & Suzuki, M. Diversification of von willebrand factor A and chitin-binding domains in Pif/BMSPs among mollusks. *J. Mol. Evol.*10.1007/s00239-024-10180-1 (2024). 38864871 10.1007/s00239-024-10180-1 PMC11291548

[79] Takeuchi, T., Sarashina, I., Iijima, M. & Endo, K. In vitro regulation of CaCO_3_ crystal polymorphism by the highly acidic molluscan shell protein Aspein. *FEBS Lett.* **582** (5), 591–596. 10.1016/j.febslet.2008.01.026 (2008). 18242173 10.1016/j.febslet.2008.01.026

[80] Addadi, L., Moradian, J., Shay, E., Maroudas, N. G. & Weiner, S. A chemical model for the cooperation of sulfates and carboxylates in calcite crystal nucleation: Relevance to biomineralization. *Proc. Natl. Acad. Sci. U. S. A.*. **84** (9), 2732–2736. 10.1073/pnas.84.9.2732 (1987). 16593827 10.1073/pnas.84.9.2732 PMC304732

[81] Maurer, P., Hohenester, E. & Engel, J. Extracellular calcium-binding proteins. *Curr. Opin. Cell Biol.* **8** (5), 609–617. 10.1016/S0955-0674(96)80101-3 (1996). 8939653 10.1016/s0955-0674(96)80101-3

[82] Pennington, K. L., Chan, T. Y., Torres, M. P. & Andersen, J. L. The dynamic and stress-adaptive signaling hub of 14-3-3: Emerging mechanisms of regulation and context-dependent protein–protein interactions. *Oncogene* **37** (42), 5587–5604. 10.1038/s41388-018-0348-3 (2018). 29915393 10.1038/s41388-018-0348-3 PMC6193947

[83] Bateman, A., Murzin, A. G. & Teichmann, S. A. Structure and distribution of pentapeptide repeats in bacteria. *Protein Sci.* **7** (6), 1477–1480. 10.1002/pro.5560070625 (1998). 9655353 10.1002/pro.5560070625 PMC2144021

[84] Vetting, M. W. et al. Pentapeptide repeat proteins. * Biochemistry* **45** (1), 1–10. 10.1021/bi052130w. (2006). 10.1021/bi052130w PMC2566302 16388575

[85] Zhang, R. & Kennedy, M. A. Current understanding of the structure and function of pentapeptide repeat proteins. *Biomolecules* **11**, (2021). 10.3390/biom11050638 PMC8145042 33925937

[86] Marin, F., Luquet, G., Marie, B. & Medakovic, D. Molluscan shell proteins: Primary structure, origin, and evolution. *Curr. Top. Dev. Biol.* **80**, 209–276 (2007). 10.1016/S0070-2153(07)80006-8 17950376

[87] Ultsch, M., Lokker, N. A., Godowski, P. J. & de Vos, A. M. Crystal structure of the NK1 fragment of human hepatocyte growth factor at 2.0 å resolution. *Structure* **6** (11), 1383–1393. 10.1016/S0969-2126(98)00138-5 (1998). 9817840 10.1016/s0969-2126(98)00138-5

[88] Bierhuizen, M. F., Mattei, M. G. & Fukuda, M. Expression of the developmental I antigen by a cloned human cDNA encoding a member of a beta-1,6-N-acetylglucosaminyltransferase gene family. *Genes Dev.* **7** (3), 468–478. 10.1101/gad.7.3.468 (1993). 8449405 10.1101/gad.7.3.468

[89] Yeh, J. C., Ong, E. & Fukuda, M. Molecular cloning and expression of a novel β-1,6-N-acetylglucosaminyltransferase that forms core 2, Core 4, and I branches*. *J. Biol. Chem.* **274** (5), 3215–3221. 10.1074/jbc.274.5.3215 (1999). 9915862 10.1074/jbc.274.5.3215

[90] Yang, Z. et al. Calcium-activated 14-3-3 proteins as a molecular switch in salt stress tolerance. *Nat. Commun.* **10** (1), 1199. 10.1038/s41467-019-09181-2 (2019). 30867421 10.1038/s41467-019-09181-2 PMC6416337

[91] Kim, C. A. & Bowie, J. U. SAM domains: Uniform structure, diversity of function. *Trends Biochem. Sci.* **28** (12), 625–628. 10.1016/j.tibs.2003.11.001 (2003). 14659692 10.1016/j.tibs.2003.11.001

[92] Kobe, B. & Kajava, A. V. The leucine-rich repeat as a protein recognition motif. *Curr. Opin. Struct. Biol.* **11** (6), 725–732. 10.1016/S0959-440X(01)00266-4 (2001). 11751054 10.1016/s0959-440x(01)00266-4

[93] Torii, K. U. Leucine-rich repeat receptor kinases in plants: Structure, function, and signal transduction pathways. *Int. Rev. Cytol.*, **234**, 1–46. (2004). 10.1016/S0074-7696(04)34001-5 15066372

[94] Mann, M. & Jensen, O. N. Proteomic analysis of post-translational modifications. *Nat. Biotechnol.* **21** (3), 255–261. 10.1038/nbt0303-255 (2003). 12610572 10.1038/nbt0303-255

[95] Levine, R. L., Moskovitz, J. & Stadtman, E. R. Oxidation of methionine in proteins: Roles in antioxidant defense and cellular regulation. *IUBMB Life*. **50** (4–5), 301–307. 10.1080/713803735 (2000). 11327324 10.1080/713803735

[96] Dimassi, S., Tabary, N., Chai, F., Blanchemain, N. & Martel, B. Sulfonated and sulfated chitosan derivatives for biomedical applications: A review. *Carbohydr. Polym.* **202**, 382–396. 10.1016/j.carbpol.2018.09.011 (2018). 30287013 10.1016/j.carbpol.2018.09.011

[97] Park, K. D. et al. Novel anti-calcification treatment of biological tissues by grafting of sulphonated poly(ethylene oxide). *Biomaterials* **18** (1), 47–51. 10.1016/S0142-9612(96)00096-8 (1997). 9003896 10.1016/s0142-9612(96)00096-8

[98] Lee, W. K. et al. Improved calcification resistance and biocompatibility of tissue patch grafted with sulfonated PEO or heparin after glutaraldehyde fixation. *J. Biomed. Mater. Res.* **58** (1), 27–35. 10.1002/1097-4636(2001)58:1%3C27::AID-JBM40%3E3.0.CO;2-2 (2001). 11152994 10.1002/1097-4636(2001)58:1<27::aid-jbm40>3.0.co;2-2

[99] Campelo, C. S. et al. In vitro evaluation of anti-calcification and anti-coagulation on sulfonated chitosan and carrageenan surfaces. *Mater. Sci. Eng. C*. **59**, 241–248. 10.1016/j.msec.2015.10.020 (2016). 10.1016/j.msec.2015.10.020 26652370

[100] Shaw, P. E. Peptidyl-prolyl isomerases: A new twist to transcription. *EMBO Rep.* **3** (6), 521–526. 10.1093/embo-reports/kvf118 (2002). 12052773 10.1093/embo-reports/kvf118 PMC1084152

[101] Nagy, P. D., Wang, R. Y., Pogany, J., Hafren, A. & Makinen, K. Emerging picture of host chaperone and cyclophilin roles in RNA virus replication. *Virology* **411** (2), 374–382. 10.1016/j.virol.2010.12.061 (2011). 21295323 10.1016/j.virol.2010.12.061

[102] Glazer, L. et al. Proteomic analysis of the crayfish gastrolith chitinous extracellular matrix reveals putative protein complexes and a central role for GAP 65. *J. Proteom.* **128**, 333–343. 10.1016/j.jprot.2015.08.016 (2015). 10.1016/j.jprot.2015.08.016 26320723

[103] Mann, K., Poustka, A. J. & Mann, M. In-depth, high-accuracy proteomics of sea urchin tooth organic matrix. *Proteome Sci.* **6** (1), 33. 10.1186/1477-5956-6-33 (2008). 19068105 10.1186/1477-5956-6-33 PMC2614417

[104] Amore, G. & Davidson, E. H. cis-Regulatory control of cyclophilin, a member of the ETS-DRI skeletogenic gene battery in the sea urchin embryo. *Dev. Biol.* **293** (2), 555–564. 10.1016/j.ydbio.2006.02.024 (2006). 16574094 10.1016/j.ydbio.2006.02.024

[105] Marie, B. et al. Different secretory repertoires control the biomineralization processes of prism and nacre deposition of the pearl oyster shell. *Proc. Natl. Acad. Sci. U S A*. **109** (51), 20986–20991. 10.1073/pnas.1210552109 (2012). 23213212 10.1073/pnas.1210552109 PMC3529032

[106] Flores, R. L. & Livingston, B. T. The skeletal proteome of the sea star Patiria miniata and evolution of biomineralization in echinoderms. *BMC Evol. Biol.* **17** (1), 125. 10.1186/s12862-017-0978-z (2017). 28583083 10.1186/s12862-017-0978-z PMC5460417

[107] Ochieng, J. & Chaudhuri, G. Cystatin superfamily. *J. Health Care Poor Underserv.*. **21** (1 Suppl), 51–70. 10.1353/hpu.0.0257 (2010). 10.1353/hpu.0.0257 PMC2888135 20173285

[108] Shen, X., Belcher, A. M., Hansma, P. K., Stucky, G. D. & Morse, D. E. Molecular cloning and characterization of lustrin A, a matrix protein from shell and pearl nacre of haliotis rufescens *. *J. Biol. Chem.* **272** (51), 32472–32481. 10.1074/jbc.272.51.32472 (1997). 9405458 10.1074/jbc.272.51.32472

[109] Kröger, N., Bergsdorf, C. & Sumper, M. A new calcium binding glycoprotein family constitutes a major diatom cell wall component. *EMBO J.* **13** (19), 4676–4683. 10.1002/j.1460-2075.1994.tb06791.x (1994). 7925309 10.1002/j.1460-2075.1994.tb06791.x PMC395402

[110] Breton, C. & Imberty, A. Structure/function studies of glycosyltransferases. *Curr. Opin. Struct. Biol.* **9** (5), 563–571. 10.1016/S0959-440X(99)00006-8 (1999). 10508766 10.1016/s0959-440x(99)00006-8

[111] Bork, P., Beckmann, G., The, C. U. B. & Domain A widespread module in developmentally regulated proteins. *J. Mol. Biol.* **231** (2), 539–545. 10.1006/jmbi.1993.1305 (1993). 8510165 10.1006/jmbi.1993.1305

[112] Kessler, E., Takahara, K., Biniaminov, L., Brusel, M. & Greenspan, D. S. Bone morphogenetic protein-1: The type I procollagen C-proteinase. *Science* **271** (5247), 360–362. 10.1126/science.271.5247.360 (1996). 8553073 10.1126/science.271.5247.360

[113] Davies, G. & Henrissat, B. Structures and mechanisms of glycosyl hydrolases. *Structure* **3** (9), 853–859. 10.1016/S0969-2126(01)00220-9 (1995). 8535779 10.1016/S0969-2126(01)00220-9

[114] Gielkens, M. et al. The abfB gene encoding the major α-L-arabinofuranosidase of Aspergillus nidulans: Nucleotide sequence, regulation and construction of a disrupted strain. *Microbiology* **145** (3), 735–741. 10.1099/13500872-145-3-735 (1999). 10217508 10.1099/13500872-145-3-735

[115] Young, J. R., Didymus, J. M., Brown, P. R., Prins, B. & Mann, S. Crystal assembly and phylogenetic evolution in heterococcoliths. *Nature* **356** (6369), 516–518. 10.1038/356516a0 (1992).

[116] Eyal, Z. et al. The variability in the structural and functional properties of coccolith base plates. *Acta Biomater.* **148**, 336–344. 10.1016/j.actbio.2022.06.027 (2022). 35738389 10.1016/j.actbio.2022.06.027

[117] Pankov, R. & Yamada, K. M. Fibronectin at a glance. *J. Cell. Sci.* **115** (20), 3861–3863. 10.1242/jcs.00059 (2002). 12244123 10.1242/jcs.00059

[118] Beckmann, G. & Bork, P. An adhesive domain detected in functionally diverse receptors. *Trends Biochem. Sci.* **18** (2), 40–41. 10.1016/0968-0004(93)90049-S (1993). 8387703 10.1016/0968-0004(93)90049-s

[119] Goldman, M. H., Pezzotti, M., Seurinck, J. & Mariani, C. Developmental expression of tobacco pistil-specific genes encoding novel extensin-like proteins. *Plant. Cell.* **4** (9), 1041–1051. 10.1105/tpc.4.9.1041 (1992). 1392607 10.1105/tpc.4.9.1041 PMC160195

[120] Hirano, T. SMC proteins and chromosome mechanics: From bacteria to humans. *Philos. Trans. R. Soc. B Biol. Sci.* **360** (1455), 507–514. 10.1098/rstb.2004.1606 (2005). 10.1098/rstb.2004.1606 PMC1569466 15897176

[121] Hadjebi, O., Casas-Terradellas, E., Garcia-Gonzalo, F. R. & Rosa, J. L. The RCC1 superfamily: From genes, to function, to disease. *Biochim. et Biophys. Acta (BBA) - Mol. Cell. Res.* **1783** (8), 1467–1479. 10.1016/j.bbamcr.2008.03.015 (2008). 10.1016/j.bbamcr.2008.03.015 18442486

[122] Seki, T., Hayashi, N. & Nishimoto, T. RCC1 in the Ran Pathway. *J. Biochem.* **120** (2), 207–214. 10.1093/oxfordjournals.jbchem.a021400 (1996). 8889801 10.1093/oxfordjournals.jbchem.a021400

[123] Yoneda, Y., Hieda, M., Nagoshi, E. & Miyamoto, Y. Nucleocytoplasmic protein transport and recycling of ran. *Cell Struct. Funct.* **24** (6), 425–433. 10.1247/csf.24.425 (1999). 10698256 10.1247/csf.24.425

[124] Vernoud, V., Horton, A. C., Yang, Z. & Nielsen, E. Analysis of the small GTPase gene superfamily of arabidopsis. *Plant Physiol.* **131** (3), 1191–1208. 10.1104/pp.013052 (2003). 12644670 10.1104/pp.013052 PMC166880

[125] Bednar, J. et al. Nucleosomes, linker DNA, and linker histone form a unique structural motif that directs the higher-order folding and compaction of chromatin. *PNAS* **95** (24), 14173–14178 (1998). 9826673 10.1073/pnas.95.24.14173 PMC24346

[126] Corstjens, P. L. A. M. et al. A calcium-binding protein in the coccolithophorid emiliania huxleyi (prymnesiophyceae). *J. Phycol.* **34** (4), 622–630. 10.1046/j.1529-8817.1998.340622.x (1998).

[127] Mackinder, L. et al. Expression of biomineralization-related ion transport genes in Emiliania huxleyi. *Environ. Microbiol.* **13** (12), 3250–3265. 10.1111/j.1462-2920.2011.02561.x (2011). 21902794 10.1111/j.1462-2920.2011.02561.x

[128] Liu, H. L. et al. Identification and characterization of a biomineralization related gene PFMG1 highly expressed in the mantle of Pinctada fucata. *Biochemistry* **46** (3), 844–851. 10.1021/bi061881a (2007). 17223706 10.1021/bi061881a

[129] Huang, J., Zhang, C., Ma, Z., Xie, L. & Zhang, R. A novel extracellular EF-hand protein involved in the shell formation of pearl oyster. *Biochim. Biophys. Acta*. **1770** (7), 1037–1044. 10.1016/j.bbagen.2007.03.006 (2007). 17451885 10.1016/j.bbagen.2007.03.006

[130] Xie, J. et al. Influence of the extrapallial fluid of pinctada fucata on the crystallization of calcium carbonate and shell biomineralization. *Cryst. Growth. Des.* **16** (2), 672–680. 10.1021/acs.cgd.5b01203 (2016).

[131] Endo, H. et al. Stable nuclear transformation system for the coccolithophorid alga pleurochrysis carterae. *Sci. Rep.* **6** (1), 22252. 10.1038/srep22252 (2016). 26947136 10.1038/srep22252 PMC4779993

[132] Perez-Riverol, Y. et al. The PRIDE database resources in 2022: A hub for mass spectrometry-based proteomics evidences. *Nucleic Acids Res.* **50** (D1), D543–D552. 10.1093/nar/gkab1038 (2022). 34723319 10.1093/nar/gkab1038 PMC8728295

