# Supplementary material for: Exploring proteins within the coccolith matrix
Source: Sci Rep. 2024 Dec 30;14:31821. doi: 10.1038/s41598-024-83052-9 (PMC11685980; doi:10.1038/s41598-024-83052-9)
Supplement: Supplementary file 1 — Supplementary Material 1 [file 41598_2024_83052_MOESM1_ESM.docx]

**Supplementary Table 2.** Proteins identified in the calcite/shell matrix of marine calcifying organisms

| **Species** | **Name**  **(Accession)** | **Structure**  **(Polymorph)** | **pI** | **% Acidic residues** | **CD-Search Conserved domain** | **Domain function** | **^Ref.^** |
| --- | --- | --- | --- | --- | --- | --- | --- |
| *Gephyrocapsa huxleyi*  Coccolithophore  **Haptophyta** | EhG6475.1 | Coccolith (calcite) | 4.19 | Asp – 9.8%  Glu – 5.7%  **15.5%** | choice-of-anchor I domain (cl45611) | Putative surface protein; collagen binding | ^1^ |
|  | Br50859.t1 | Coccolith (calcite) | 4.31 | Asp – 8.6%  Glu – 12.7%  **21.3%** | D-arabinose 1-dehydrogenase, Zn-dependent alcohol dehydrogenase family (cl43322) | Carbohydrate metabolism | ^1^ |
|  | EhG17242.1 | Coccolith (calcite) | 5.17 | Asp – 5.9%  Glu – 6.5%  **12.4%** | None |  | ^1^ |
|  | EhG17242.7 | Coccolith (calcite) | 4.72 | Asp – 5.2%  Glu – 8.1%  **13.3%** | None |  | ^1^ |
|  | EhG21037.1 | Coccolith (calcite) | 5.12 | Asp – 9.4%  Glu – 3.5%  **12.9%** | Uncharacterized protein YjbI, contains pentapeptide repeats (COG1357) | Exact function unknown | ^1^ |
|  | EhG21537.1 | Coccolith (calcite) | 9.87 | Asp – 3.8%  Glu – 3.2%  **7.0%** | None |  | ^1^ |
|  | EhG30161.1 | Coccolith (calcite) | 5.62 | Asp – 3.9%  Glu – 2.6%  **6.5%** | Uncharacterized protein YjbI, contains pentapeptide repeats (COG1357) | Exact function unknown | ^1^ |
|  | EhG5157.1 | Coccolith (calcite) | 5.40 | Asp – 4.1%  Glu – 4.1%  **8.2%** | Uncharacterized protein YjbI, contains pentapeptide repeats (COG1357) | Exact function unknown | ^1^ |
|  | EhG17918.2 | Coccolith (calcite) | 4.89 | Asp – 3.8%  Glu – 4.3%  **8.1%** | None |  | ^1^ |
|  | EhG20254.1 | Coccolith (calcite) | 4.48 | Asp – 6.4%  Glu – 5.0%  **11.4%** | None |  | ^1^ |
|  | EhG42278.1 | Coccolith (calcite) | 4.52 | Asp – 5.5%  Glu – 5.2%  **10.7%** | None |  | ^1^ |
|  | EhG28195.1 | Coccolith (calcite) | 5.71 | Asp – 1.8%  Glu – 7.5%  **9.3%** | None |  | ^1^ |
|  | EhG40669.2 | Coccolith (calcite) | 5.42 | Asp – 4.6%  Glu - 3.8%  **8.4%** | Uncharacterized protein YjbI, contains pentapeptide repeats (COG1357) | Exact function unknown | ^1^ |
|  | EhG41650.1 | Coccolith (calcite) | 4.61 | Asp – 5.8%  Glu – 7.2%  **13.0%** | None |  | ^1^ |
|  | EhG42557.1 | Coccolith (calcite) | 8.05 | Asp – 2.0%  Glu – 8.9%  **10.9%** | None |  | ^1^ |
|  | Br844.t1 | Coccolith (calcite) | 8.48 | Asp – 5.9%  Glu – 3.6%  **9.5%** | None |  | ^1^ |
|  | Br27222.t1 | Coccolith (calcite) | 8.10 | Asp – 5.3%  Glu – 3.2%  **8.5%** | None |  | ^1^ |
|  | EhG4764.1 | Coccolith (calcite) | 4.91 | Asp – 6.7%  Glu – 2.0%  **9.7%** | Mycoplasma protein of unknown function, DUF285 (pfam03382) | Exact function unknown | ^1^ |
|  | EhG4774.4 | Coccolith (calcite) | 7.04 | Asp – 6.8%  Glu – 1.7%  **8.5%** | Mycoplasma protein of unknown function, DUF285 (pfam03382) | Exact function unknown | ^1^ |
|  | EhG34604.1 | Coccolith (calcite) | 4.82 | Asp – 8.3%  Glu – 1.3%  **9.6%** | Mycoplasma protein of unknown function, DUF285 (pfam03382) X2 | Exact function unknown | ^1^ |
|  | EhG26439.1 | Coccolith (calcite) | 6.02 | Asp – 4.9%  Glu – 3.5%  **8.4%** | Uncharacterized protein YjbI, contains pentapeptide repeats (COG1357) | Exact function unknown | ^1^ |
|  | Br15983.t1 | Coccolith (calcite) | 6.19 | Asp – 4.6%  Glu – 3.4%  **8.0%** | Uncharacterized protein YjbI, contains pentapeptide repeats (COG1357) | Exact function unknown | ^1^ |
|  | EhG32342.1 | Coccolith (calcite) | 6.46 | Asp – 2.7%  Glu – 4.2%  **6.9%** | Alpha-tubulin suppressor and related RCC1 domain-containing proteins (cl34932) | Cytoskeleton, Cell cycle control, cell division, chromosome partitioning | ^1^ |
|  | EhG33272.1 | Coccolith (calcite) | 6.75 | Asp – 3.0%  Glu – 2.5%  **5.5%** | MAM domain (cd06263) | Extracellular domain, protein-protein interaction, cell adhesion | ^1^ |
|  | EhG38955.4 | Coccolith (calcite) | 4.49 | Asp – 6.5%  Glu – 5.3%  **11.8%** | None |  | ^1^ |
| *Crassostrea nippona*  (Iwagaki oyster)  **Mollusca** | Nacrein-like protein C1  (A0ZSF6.1) | NA | 6.01 | Asp – 6.2%  Glu – 7.9%  **14.1%** | Carbonic anhydrase alpha (cd00326) | Reversible hydration of carbon dioxide to bicarbonate | ^2^ |
|  | Nacrein-like protein C2  (A0ZSF7.1) | NA | 6.85 | Asp – 6.0%  Glu – 7.2%  **13.2%** | Carbonic anhydrase alpha (cd00326) | Reversible hydration of carbon dioxide to bicarbonate | ^2^ |
| *Crassostrea gigas*  (Pacific oyster)  **Mollusca** | Pif97  (AFT63505.1) | Calcite | 8.28 | Asp – 5.3%  Glu – 5.3%  **10.6%** | Von Willebrand factor type A (cd01450);  Laminin G domain (cl22861) | Multiple cellular processes, ligand binding;  Ca^2+^-mediated sensor, ligand binding | ^3^ |
|  | Nacrein-like proteins F1  (AGN98077.1) | NA | 4.87 | Asp – 9.1%  Glu – 9.3%  **18.4%** | Eukaryotic-type carbonic anhydrase (pfam00194) | Reversible hydration of carbon dioxide to bicarbonate | ^4^ |
|  | Nacrein-like proteins F2  AGN98076.1) | NA | 6.35 | Asp – 8.2%  Glu – 8.2%  **16.4%** | Eukaryotic-type carbonic anhydrase (pfam00194) | Reversible hydration of carbon dioxide to bicarbonate | ^4^ |
|  | Nacrein-like proteins F3  (AGN98078.1) | NA | NA | Asp – 9.7%  Glu – 9.0%  **18.7%** | Eukaryotic-type carbonic anhydrase (smart01057) | Reversible hydration of carbon dioxide to bicarbonate | ^4^ |
| *Haliotis laevigata*  Greenlip abalone  **Mollusca** | Perlustrin  (P82595) | Nacre (aragonite) | 8.02 | Asp – 3.6%  Glu – 4.8%  **8.4%** | Insulin growth factor-binding protein homologues (smart00121) | High affinity insulin-like growth factors binding | ^5^ |
|  | Perlucin  (P82596) | Nacre (aragonite) | 7.15 | Asp – 4.5%  Glu – 6.5%  **11.0%** | C-type lectin (CTL)/C-type lectin-like (CTLD) domain (cl02432) | Ca^2+^-mediated ligand binding, commonly carbohydrates | ^6^ |
| *Haliotis rufescens*  Red abalone  **Mollusca** | Lustrin A  (AAB95154.1) | Nacre (aragonite) | 8.12 | Asp – 3.9%  Glu – 2.1%  **6.0%** | WAP-type (Whey Acidic Protein) 'four-disulfide core' (pfam00095) | Protease inhibition | ^7^ |
|  | AP7  (AAK00635.1) | Nacre (aragonite) | 5.46 | Asp – 6.8%  Glu – 3.4%  **10.2%** | None |  | ^8^ |
|  | AP24  (AAK00634.1) | Nacre (aragonite) | 5.78 | Asp – 6.4%  Glu – 5.3%  **11.7%** | None |  | ^8^ |
| *Mizuhopecten*  *yessoensis*  Yesso scallop  **Mollusca** | MSP-1  (Q95YF6) | Foliated (calcite) | 3.36 | Asp – 19.6%  Glu – 2.6%  **22.2%** | None |  | ^9^ |
|  | Nacrein-like protein P1 (A0ZSF4.1) | NA | 6.01 | Asp – 6.3%  Glu – 8.2%  **14.5%** | Carbonic anhydrase alpha (cd00326) | Reversible hydration of carbon dioxide to bicarbonate | ^2^ |
|  | Nacrein-like protein P2 (A0ZSF5.1) | NA | 6.60 | Asp – 6.5%  Glu – 6.7%  **13.2%** | Carbonic anhydrase alpha, prokaryotic-like subfamily (cd03124) | Reversible hydration of carbon dioxide to bicarbonate | ^2^ |
| *Mytilus*  *galloprovincialis*  Mediterranean mussel  **Mollusca** | BMSP  (BAK86420.1) | Aragonite & calcite | 8.92 | Asp – 5.7%  Glu – 3.7%  **9.4%** | von Willebrand factor (vWF) type A domain (smart00327) X2;  von Willebrand factor type A domain (pfam00092) X2;  Chitin-binding domain type 2 (smart00494);  Chitin binding Peritrophin-A domain (pfam01607) | Multiple cellular processes, ligand binding;  Chitin binding | ^10^ |
| *Patella vulgate*  Common limpet  **Mollusca** | BMSP protein  (CCJ09597) | NA | 4.61 | Asp – 6.9%  Glu – 4.6%  **11.5%** | von Willebrand factor (vWF) type A domain (smart00327);  Chitin binding Peritrophin-A domain (pfam01607) X2 | Multiple cellular processes, ligand binding;  Chitin binding | ^11^ |
| *Pinctada fucata*  Akoya pearl oyster  **Mollusca** | Nacrein  (Q27908) | Nacre (aragonite) | 6.83 | Asp – 6.3%  Glu – 6.3%  **12.6%** | Carbonic anhydrase alpha, prokaryotic-like subfamily (cd03124) | Reversible hydration of carbon dioxide to bicarbonate | ^12^ |
|  | Nacrein-like protein F  (A0ZSF2.1) | Nacre (aragonite) | 7.75 | Asp – 6.0%  Glu – 7.0%  **13.0%** | Carbonic anhydrase alpha (cd00326) | Reversible hydration of carbon dioxide to bicarbonate | ^13^ |
|  | N16/Pearlin  (O97048) | Nacre (aragonite) | 6.10 | Asp – 7.8%  Glu – 6.2%  **14.0%** | None |  | ^14^ |
|  | MSI 60  (O02402) | Nacre (aragonite) | 4.88 | Asp – 5.4%  Glu – 0.4%  **5.8%** | None |  | ^15^ |
|  | MSI 31/Glycine-rich protein  (O02401) | Prisms (calcite) | 3.82 | Asp – 5.1%  Glu – 8.1%  **13.2%** | None |  | ^15^ |
|  | MSI 7/ Glycine-rich shell matrix protein  (Q7YWA5) | Prisms (calcite) | 6.69 | Asp -2.1%  Glu – 0.0%  **2.1%** | None |  | ^16^ |
|  | Aspein  (BAD00044) | Prisms (calcite) | 1.97 | 57.6%  1.5%  **59.1%** | None |  | ^17^ |
|  | Prismalin-14  (BAD27406) | Prisms | 4.32 | Asp – 9.1%  Glu – 0.0%  **9.1%** | None |  | ^18^ |
|  | Pif177  (BAH97338.1) | Nacre (aragonite) | 4.99 | Asp – 20.8%  Glu – 5.3%  **26.3%** | von Willebrand factor (vWF) type A domain (smart00327) | Multiple cellular processes, ligand binding | ^19^ |
|  | KRMP-1/ lysine-rich matrix protein-1  (AAZ95764.1) | Prisms (calcite) | 9.55 | Asp – 5.1%  Glu – 0.0%  **5.1%** | None |  | ^20^ |
|  | KRMP-2/ lysine-rich matrix protein-2  (AAZ95764.1) | Prisms (calcite) | 9.41 | Asp – 5.0%  Glu – 0.0%  **5.0%** | None |  | ^20^ |
|  | KRMP-3/ lysine-rich matrix protein-3  (AAZ95765.1) | Prisms (calcite) | 9.41 | Asp – 5.0%  Glu – 0.0%  **5.0%** | None |  | ^20^ |
|  | PfN44  (AGG35567.1) | Nacre (aragonite) | 4.29 | Asp – 6.4%  Glu – 3.0%  **9.4%** | von Willebrand factor type C domain (cl17735) | Multiple cellular processes, ligand binding | ^21^ |
|  | PFMG1  (AAZ76255.1) | NA | 7.69 | Asp – 8.8%  Glu – 8.1%  **16.9%** | EF-hand, calcium binding motif, found in CREC-EF hand family (cl25354) | Low-affinity Ca^2+^ binding, secretory pathway, chaperone activity, signal transduction | ^22^ |
|  | EFCBP  (ABF48089.1) | Nacre (aragonite) | 8.99 | Asp – 10.9%  Glu – 4.7%  **15.6%** | EF-hand, calcium binding motif (cl08302) | Ca^2+^ binding | ^23^ |
|  | Shematrin-1  (BAE93433.1) | Prisms (calcite) | 9.13 | Asp – 0.3%  Glu – 0.0%  **0.3%** | None |  | ^24^ |
|  | Shematrin-2  (BAE93434.1) | Prisms (calcite) | 9.42 | Asp – 1.0%  Glu – 0.0%  **1.0%** | None |  | ^24^ |
|  | Shematrin-3  (BAE93435.1) | Prisms (calcite) | 9.36 | Asp – 0.0%  Glu – 0.0%  **0.0%** | None |  | ^24^ |
|  | Shematrin-4  (BAE93436.1) | Prisms (calcite) | 9.17 | Asp – 0.7%  Glu – 0.0%  **0.7%** | DEAD-like_helicase_N super family (cl28899) | ATP-dependent RNA/DNA unwinding | ^24^ |
|  | Shematrin-5  (BAE93437.1) | Prisms (calcite) | 7.45 | Asp – 10.1%  Glu – 1.1%  **11.2%** | None |  | ^24^ |
|  | Shematrin-6  (BAE93438.1) | Prisms (calcite) | 9.67 | Asp – 0.7%  Glu – 0.0%  **0.7%** | None |  | ^24^ |
|  | Shematrin-7  (BAE93439.1) | Prisms (calcite) | 10.21 | Asp – 0.3%  Glu – 1.0%  **1.3%** | None |  | ^24^ |
|  | PfY2  (ASM90391.1) | NA | 8.94 | Asp – 2.7%  Glu – 1.8%  **4.5%** | None |  | ^25^ |
|  | ACCBP  (ABF13208.1) | NA | 4.72 | Asp – 10.0%  Glu – 4.6%  **14.6%** | extracellular domain (ECD) of ligand-gated ion channel (LGIC) (cl28912) | Extracellular ion channel | ^26^ |
|  | SPARC  (AND99565.1) | NA | 5.36 | Asp – 11.6%  Glu – 7.1%  **18.7%** | EF-hand, extracellular calcium-binding (EC) motif, found in secreted protein acidic and rich in cysteine (SPARC)-like proteins (cl25349);  Kazal type serine protease inhibitors and follistatin-like domains (cl00097) | Extracellular calcium binding, cell-environment interaction, extracellular matrix formation/deposition, counter-adhesion, protease regulation, cell signalling, cell development;  Serine protease inhibitor | ^27^ |
| *Pinctada maxima*  Silver lip pearl oyster  **Mollusca** | N66  (BAA90540.1) | Nacre (aragonite) | 8.68 | Asp – 5.5%  Glu – 4.4%  **9.9%** | Carbonic anhydrase alpha (vertebrate-like) group (cl00012) X2 | Reversible hydration of carbon dioxide to bicarbonate | ^28^ |
|  | N45  (ACT55367.1) |  | 8.80 | Asp – 6.5%  Glu – 4.9%  **11.4%** | Carbonic anhydrase alpha, prokaryotic-like subfamily (cd03124) | Reversible hydration of carbon dioxide to bicarbonate | ^29^ |
|  | N36  (ACS50182.1) |  | 6.09 | Asp – 6.7%  Glu – 6.1%  **12.8%** | Carbonic anhydrase alpha (vertebrate-like) group (cl00012) | Reversible hydration of carbon dioxide to bicarbonate | ^29^ |
|  | Aspein  (BAL21566.1) | Prisms (calcite) | 1.79 | Asp – 75.5%  Glu – 0.4%  **75.9%** | None |  | ^30^ |
|  | N14  (BAA90539.1) | Nacre (aragonite) | 5.40 | Asp – 7.1%  Glu – 5.7%  **12.8%** | None |  | ^28^ |
|  | Nacrein like protein  (BAF42330.1) | Nacre | 6.72 | Asp – 6.7%  Glu – 6.9%  **13.6%** | Carbonic anhydrase alpha, prokaryotic-like subfamily (cd03124) | Reversible hydration of carbon dioxide to bicarbonate | ^2^ |
|  | Pif  (BAJ08001.1) | Nacre (aragonite) | 4.84 | Asp – 19.7%  Glu – 5.6%  **25.3%** | von Willebrand factor type A domain (pfam00092) | Multiple cellular processes, ligand binding | ^31^ |
|  | Shematrin-1a  (AGG15945.1) | Prisms (calcite) | 9.04 | Asp – 0.6%  Glu – 0.0%  **0.6%** | None |  | ^32^ |
|  | Shematrin-1b  (AGG15946.1) | Prisms (calcite) | 9.22 | Asp – 0.3%  Glu – 0.0%  **0.3%** | None |  | ^32^ |
|  | Shematrin-2a  (AGG15947.1) | Prisms (calcite) | 9.28 | Asp – 1.0%  Glu – 0.0%  **1.0%** | None |  | ^32^ |
|  | Shematrin-2b  (AGG15940.1) | Prisms (calcite) | 9.84 | Asp – 0.0%  Glu – 0.0%  **0.0%** | None |  | ^32^ |
|  | Shematrin-3  (AGG15948.1) | Prisms (calcite) | 9.44 | Asp – 0.3%  Glu – 0.0%  **0.3%** | None |  | ^32^ |
|  | Shematrin-4  (AGG15941.1) | Prisms (calcite) | 8.83 | Asp – 0.5%  Glu – 0.0%  **0.5%** | DEAD-like_helicase_N super family (cl28899) | ATP-dependent RNA/DNA unwinding | ^32^ |
|  | Shematrin-5  (AGG15942.1) | Prisms (calcite) | 9.82 | Asp – 1.2%  Glu – 0.0%  **1.2%** | None |  | ^32^ |
|  | Shematrin-6  (AGG15943.1) | Prisms (calcite) | 9.56 | Asp – 0.3%  Glu – 0.0%  **0.3%** | None |  | ^32^ |
|  | Shematrin-7 (AGG15944.1) | Prisms (calcite) | 9.86 | Asp – 0.3%  Glu – 1.0%  **1.3%** | This family represents the protein UNC80 found in eukaryotes, a component of the NALCN sodium channel complex (cl44845) | Sodium channel | ^32^ |
| *Pinctada margaritifera*  Black lip pearl oyster  **Mollusca** | Nacrein A1  (AEC03970.1) | Nacre (aragonite) | 8.74 | Asp – 5.2%  Glu – 4.3%  **8.5%** | Carbonic anhydrase alpha (vertebrate-like) group (cl00012) X2 | Reversible hydration of carbon dioxide to bicarbonate | ^33^ |
|  | Nacrein B2  (ADY69618.1) | Nacre (aragonite) | 7.90 | Asp – 6.1%  Glu – 4.9%  **11.0%** | Carbonic anhydrase alpha (vertebrate-like) group (cl00012) X2 | Reversible hydration of carbon dioxide to bicarbonate | ^33^ |
|  | Nacrein B3  (AEC03971.1) | Nacre (aragonite) | 8.82 | Asp – 5.7%  Glu – 4.3%  **10.0%** | Carbonic anhydrase alpha (vertebrate-like) group (cl00012) X2 | Reversible hydration of carbon dioxide to bicarbonate | ^33^ |
|  | Nacrein B4  (AEC03972.1) | Nacre (aragonite) | 8.38 | Asp – 5.9%  Glu – 4.5%  **10.4%** | Carbonic anhydrase alpha (vertebrate-like) group (cl00012) X2 | Reversible hydration of carbon dioxide to bicarbonate | ^33^ |
|  | Nacrein C5  (AEC03973.1) | Nacre (aragonite) | 8.81 | Asp – 6.6%  Glu – 5.5%  **12.1%** | Carbonic anhydrase alpha (cd00326) | Reversible hydration of carbon dioxide to bicarbonate | ^33^ |
|  | Pif  (BAM66823.1) | Nacre (aragonite) | 4.94 | Asp – 19.6%  Glu - 5.9%  **25.5%** | von Willebrand factor (vWF) type A domain (smart00327);  PTZ00121 super family (cl31754) | Multiple cellular processes, ligand binding;  Unknown | ^31^ |
| *Pinna nobilis*  Fan mussel  **Mollusca** | Mucoperlin  (AAK18045.1) | Nacre (aragonite) | 4.87 | Asp – 4.7%  Glu – 4.6%  **9.3%** | None |  | ^34^ |
| *Stylophora pistillata*  Scleractinian coral  **Cnidaria** | Protocadherin fat-like  (AGG36361.1) | Skeletal organic matrix (SOM) | 4.88 | Asp – 7.3%  Glu – 7.1%  **14.4%** | Cadherin tandem repeat domain (cd11304) X13 | Glycoprotein involved in Ca^2+^-mediated cell-cell adhesion | ^35^ |
|  | CARP8  (AGG36357.1) | Skeletal organic matrix (SOM) | 3.91 | Asp – 23.2%  Glu – 6.1%  **29.3%** | None |  | ^35^ |
|  | Thrombspondin  (AGG36335.1) | Skeletal organic matrix (SOM) | 5.45 | Asp – 5.4%  Glu – 7.5%  **12.9%** | Von Willebrand factor type A (vWA) domain (cd01450);  Thrombospondin type 1 repeats (smart00209) | Multiple cellular processes, ligand binding;  TGF-beta activation | ^35^ |
|  | Viral inclusion protein  (AGG36336.1) | Skeletal organic matrix (SOM) | 5.34 | Asp – 7.8%  Glu – 13.1%  **20.9%** | Chromosome segregation ATPase (cl34174) X3;  Chromosome segregation protein SMC, common bacterial type (cl37069) X2 | Cell cycle, cell division | ^35^ |
|  | Hemicentrin  (AGE45658.1) | Skeletal organic matrix (SOM) | 4.83 | Asp – 3.5%  Glu – 5.4%  **8.9%** | Von Willebrand factor type A (vWA) domain (smart00327);  Thrombospondin type 1 repeats (smart00209) X2 | Multiple cellular processes, ligand binding;  TGF-beta activation | ^35^ |
|  | Actin  (AGE45656.1) | Skeletal organic matrix (SOM) | 5.78 | Asp – 4.7%  Glu – 7.8%  **12.5%** | Nucleotide-Binding Domain of the sugar kinase/HSP70/actin superfamily (cl17037) | Multiple | ^35^ |
|  | Actin  (AGG36337.1) | Skeletal organic matrix (SOM) | 5.30 | Asp – 5.6%  Glu – 7.4%  **13.0%** | actin; Provisional (PTZ00281) | Cytoskeleton | ^35^ |
|  | Major yolk protein  (AGG36338.1) | Skeletal organic matrix (SOM) | 7.07 | Asp – 7.8%  Glu – 6.0%  **13.8%** | Transferrin family of the type 2 periplasmic-binding protein superfamily (cd13529);  Type 2 periplasmic binding fold superfamily (cl21456);  Transferrin (cl30084) | Metal-binding (typically Fe) glycoprotein;  Ligand-binding domain serving as initial receptor for transport, signal transduction and channel gating | ^35^ |
|  | Protocadherin fat-like  (AGC70194.1) | Skeletal organic matrix (SOM) | 5.35 | Asp – 7.3%  Glu – 4.2%  **11.5%** | Cadherin tandem repeat domain (cd11304) X7 | Glycoprotein involved in Ca^2+^-mediated cell-cell adhesion | ^35^ |
|  | Cadherin  (AGG36360.1) | Skeletal organic matrix (SOM) | 4.78 | Asp – 6.9%  Glu – 5.6%  **12.5%** | Cadherin tandem repeat domain (cd11304) X4;  Laminin G domain (cd00110);  Calcium-binding EGF-like domain (cd00054);  Band 3 cytoplasmic domain (cl26877) | Glycoprotein involved in Ca^2+^-mediated cell-cell adhesion;  Ca^2+^-mediated sensor, ligand binding;  Ca^2+^-dependent membrane-bound or extracellular protein, protein-protein interaction;  Chloride/bicarbonate channel | ^35^ |
|  | Actin  (AGG36339.1) | Skeletal organic matrix (SOM) | 5.30 | Asp – 5.3%  Glu – 7.5%  **12.8%** | Nucleotide-Binding Domain of the sugar kinase/HSP70/actin superfamily (cl17037) | Multiple | ^35^ |
|  | Hypothetical protein  (AGC24391.1) | Skeletal organic matrix (SOM) | 5.94 | Asp – 5.1%  Glu – 5.6%  **10.7%** | None |  | ^35^ |
|  | Sushi-domain protein  (AGG36340.1) | Skeletal organic matrix (SOM) | 5.12 | Asp – 5.7%  Glu – 4.8%  **10.5%** | von Willebrand factor type D domain (cl02516);  AMOP domain (cl02758);  Nidogen-like (pfam06119) | Multiple cellular processes, ligand binding;  Cell adhesion;  Collagen-binding, cell-extracellular matrix interaction | ^35^ |
|  | Collagen – alpha  (AGG36341.1) | Skeletal organic matrix (SOM) | 5.07 | Asp – 5.5%  Glu – 6.6%  **12.1%** | Von Willebrand factor type A (vWA) domain (cl00057) | Multiple cellular processes, ligand binding; | ^35^ |
|  | CARP5  (AGG36358.1) | Skeletal organic matrix (SOM) | 4.04 | Asp – 29.1%  Glu – 7.4%  **36.5%** | None |  | ^35^ |
|  | Hypothetical protein  (AGG36342.1) | Skeletal organic matrix (SOM) | 5.02 | Asp – 11.1%  Glu – 5.4%  **16.5%** | None |  | ^35^ |
|  | Glyceraldehyde 3-phosphatase dehydrogenase  (AGE45657.1) | Skeletal organic matrix (SOM) | 9.05 | Asp – 5.8%  Glu – 4.2%  **10.0%** | glyceraldehyde-3-phosphate dehydrogenase (cl30355) | Glycolysis/gluconeogenesis, carbohydrate metabolism | ^35^ |
|  | Collagen – alpha  (AGG36343.1) | Skeletal organic matrix (SOM) | 9.07 | Asp – 3.8%  Glu – 4.8%  **8.6%** | Thrombospondin type 1 repeats (smart00209);  von Willebrand factor type A domain (pfam00092);  Von Willebrand factor (vWF) type A domain (smart00327) | TGF-beta activation;  Multiple cellular processes, ligand binding | ^35^ |
|  | Contactin-associated protein  (AGG36344.1) | Skeletal organic matrix (SOM) | 6.58 | Asp – 5.3%  Glu – 7.7%  **13.0%** | Laminin G domain (pfam02210) | Ca^2+^-mediated sensor, ligand binding; | ^35^ |
|  | MAM domain anchor protein  (AGG36345.1) | Skeletal organic matrix (SOM) | 9.26 | Asp – 4.7%  Glu - 2.9%  **7.6%** | MAM domain (cd06263);  MAM super family (cl27660);  Trefoil (P-type) domain (pfam00088) | Extracellular domain, protein-protein interaction, cell adhesion;  Mucin-associated molecule | ^35^ |
|  | Zona pellucida  (AGG36346.1) | Skeletal organic matrix (SOM) | 5.04 | Asp – 7.1%  Glu – 8.2%  **15.3%** | Zona pellucida-like domain (pfam00100) | Receptor-like glycoprotein | ^35^ |
|  | Hypothetical protein  (AGG36359.1) | Skeletal organic matrix (SOM) | 5.45 | Asp – 5.2%  Glu – 4.9%  **10.1%** | None |  | ^35^ |
|  | myosin regulatory light chain-like protein  (AGC70196.1) | Skeletal organic matrix (SOM) | 6.01 | Asp – 6.4%  Glu – 3.1%  **9.5%** | Copper/zinc superoxide dismutase (SODC) (pfam00080) | Defence against oxidative stress | ^35^ |
|  | Vitellogenin  (AGG36347.1) | Skeletal organic matrix (SOM) | 6.17 | Asp – 6.1%  Glu – 8.3%  **14.4%** | Domain of unknown function (DUF1943) (cl11721) | Exact function unknown | ^35^ |
|  | Ubiquitin  (AGG36348.1) | Skeletal organic matrix (SOM) | 7.05 | Asp – 6.6%  Glu – 7.9%  **14.5%** | ubiquitin-like (Ubl) domain (cd01803) X5;  Ubl1_cv_Nsp3_N-like super family (cl28922) | Protein modification | ^35^ |
|  | Vitellogenin  (AGG36349.1) | Skeletal organic matrix (SOM) | 6.63 | Asp – 6.5%  Glu – 6.5%  **13.0%** | None |  | ^35^ |
|  | Integrin - alpha  (AGG36350.1) | Skeletal organic matrix (SOM) | 4.52 | Asp – 4.8%  Glu – 10.2%  **15.0%** | None |  | ^35^ |
|  | Late embryogenesis protein  (AGE45655.1) | Skeletal organic matrix (SOM) | 6.05 | Asp – 1.2%  Glu – 19.6%  **20.8%** | MAEBL (cl31754) | Exact function unknown | ^35^ |
|  | Tubulin - beta  (AGG36351.1) | Skeletal organic matrix (SOM) | 5.13 | Asp – 5.6%  Glu – 6.9%  **12.5%** | tubulin beta chain (cl30499) | Cytoskeleton, cell cycle, cell division | ^35^ |
|  | Protocadherin (AGC70195.1) | Skeletal organic matrix (SOM) | 4.81 | Asp – 8.4%  Glu – 4.6%  **13.0%** | Cadherin tandem repeat domain (cd11304) X25 | Glycoprotein involved in Ca^2+^-mediated cell-cell adhesion | ^35^ |
|  | Neurexin  (AGG36352.1) | Skeletal organic matrix (SOM) | 8.73 | Asp – 7.6%  Glu – 3.2%  **10.8%** | Laminin G domain (smart00282);  Laminin G domain (cd00110);  EGF-like domain (pfam00008) | Ca^2+^-mediated sensor, ligand binding;  Ca^2+^-dependent membrane-bound or extracellular protein, protein-protein interaction; | ^35^ |
|  | Kielin/chordin llke  (AGG36353.1) | Skeletal organic matrix (SOM) | 8.37 | Asp – 7.3%  Glu – 3.8%  **11.1%** | von Willebrand factor type C domain (pfam00093);  Trypsin Inhibitor like cysteine rich domain (pfam01826) | Multiple cellular processes, ligand binding  Trypsin inhibitor | ^35^ |
|  | Flagellar associated protein  (AGG36354.1) | Skeletal organic matrix (SOM) | 5.90 | Asp – 6.9%  Glu – 7.7%  **14.6%** | None |  | ^35^ |
|  | Carbonic anhydrase (STPCA2)  (ACE95141.1) | Skeletal organic matrix (SOM) | 6.99 | Asp – 6.3%  Glu – 6.0%  **12.3%** | Eukaryotic-type carbonic anhydrase (pfam00194) | Reversible hydration of carbon dioxide to bicarbonate | ^35^ |
|  | Zonadhesion-like precursor  (AGG36356.1) | Skeletal organic matrix (SOM) | 8.28 | Asp – 5.3%  Glu – 3.3%  **8.6%** | MAM domain (cd06263) X2 | Extracellular domain, protein-protein interaction, cell adhesion | ^35^ |
| *Acropora millepora*  Scleractinian coral  **Cnidaria** | Mucin-like protein  (B3EWY9) | Skeletal organic matrix (SOM) | 5.90 | Asp – 5.9%  Glu – 4.5%  **10.4%** | Nidogen-like domain (cl02648);  Thrombospondin type 1 repeats (smart00209) X3;  von Willebrand factor type D domain (cl02516);  Calcium-binding EGF-like domain (smart00179) X4;  Complement Clr-like EGF-like (pfam12662);  Coagulation Factor Xa inhibitory site (pfam14670);  AMOP domain (cl02758) | Extracellular domain;  TGF-beta activation;  Multiple cellular processes, ligand binding;  Ca^2+^-dependent membrane-bound or extracellular protein, protein-protein interaction | ^36^ |
|  | Coadhesin  (B3EWZ3) | Skeletal organic matrix (SOM) | 5.77 | Asp – 5.6%  Glu – 5.2%  **10.8%** | von Willebrand factor (vWF) type A domain (smart00327);  Von Willebrand factor type A (vWA) domain (cd01450);  von Willebrand factor type A domain (pfam00092)  Coagulation factor 5/8 C-terminal domain (cl25480) X2;  Thrombospondin type 1 repeats (smart00209) X8;  CASIMO1 super family (cl24410) | Multiple cellular processes, ligand binding;  TGF-beta activation | ^36^ |
|  | Ectin  (B3EWZ8) | Skeletal organic matrix (SOM) | 4.62 | Asp – 5.4%  Glu – 7.0%  **12.4%** | CAP_GAPR1-like domain (cd05382);  Thrombospondin type 1 repeats (smart00209) X2 | Golgi-associated plant pathogenesis related protein 1;  TGF-beta activation | ^36^ |
|  | MAM and Fibronectin-containing protein  (B3EX02) | Skeletal organic matrix (SOM) | 7.25 | Asp – 3.8%  Glu – 4.3%  **8.1%** | MAM domain (cd06263);  Fibronectin type 3 domain (cd00063);  Fibronectin type III domain (pfam00041) | Extracellular domain, protein-protein interaction, cell adhesion;  Multiple | ^36^ |
|  | Zona pellucida domain-containing protein  (G8HTB6) | Skeletal organic matrix (SOM) | 4.92 | Asp – 6.5%  Glu – 6.8%  **13.3%** | Zona pellucida-like domain (pfam00100) | Receptor-like glycoprotein | ^36^ |
|  | CUB domain-containing protein  (B3EX01) | Skeletal organic matrix (SOM) | 4.99 | Asp – 5.4%  Glu – 6.4%  **11.8%** | CUB domain (cd00041);  Ly-6 antigen / uPA receptor -like domain (cl10471) | Extracellular domain, typically involved in development;  GPI-linked cell-surface glycoprotein | ^36^ |
|  | Polycystin-1-related-related protein  (B8UU59) | Skeletal organic matrix (SOM) | 7.48 | Asp – 4.7%  Glu – 5.5%  **10.2%** | PLAT (Polycystin-1, Lipoxygenase, Alpha-Toxin) domain (cl00011);  REJ domain (cl28747);  Polycystin cation channel protein (cl28216);  Polycystin cation channel (cl37568);  GPCR proteolysis site (cl02559);  WSC domain (cl02568) | Access to membrane- or micelle- bound substrates;  Extracellular domain of unknown exact function;  Cation transport;  Cell adhesion auto-proteolysis;  Carbohydrate binding | ^36^ |
|  | EGF and laminin G domain-containing protein  (B8UU78) | Skeletal organic matrix (SOM) | 6.77 | Asp – 5.9%  Glu – 6.4%  **11.3%** | Laminin G domain (pfam02210);  Laminin G domain (cd00110) X2;  Laminin G domain (cl22861) | Ca^2+^-mediated sensor, ligand binding | ^36^ |
|  | Protocadherin-like protein  (B8V7Q1) | Skeletal organic matrix (SOM) | 5.00 | Asp – 7.3%  Glu – 5.5%  **12.8%** | Cadherin tandem repeat domain (cd11304) X28;  Cadherin cytoplasmic region (cl03079);  Laminin G domain (cd00110);  Laminin G domain (smart00282);  Calcium-binding EGF-like domain (cd00054);  EGF-like domain (pfam00008) | Glycoprotein involved in Ca^2+^-mediated cell-cell adhesion;  Ca^2+^-mediated sensor, ligand binding;  Ca^2+^-dependent membrane-bound or extracellular protein, protein-protein interaction; | ^36^ |
|  | Collagen alpha  (B8V7R6) | Skeletal organic matrix (SOM) | 5.62 | Asp – 5.9%  Glu – 6.2%  **12.1%** | Fibrillar collagen C-terminal domain (cl02436);  LPXTG-anchored collagen-like adhesin Scl2/SclB (cl45768) X2 | Intracellular molecular assembly and extracellular formation of collagen fibrils;  Adhesion | ^36^ |
|  | Neuroglian-like protein  (B8VIW9) | Skeletal organic matrix (SOM) | 5.70 | Asp – 5.2%  Glu – 7.3%  **12.5%** | Bravo-like intracellular region (pfam13882);  Fibronectin type 3 domain (cd00063) X4;  Immunoglobulin domain (cl11960) X3;  Immunoglobulin domain (cd00096) | Adhesion;  Multiple;  Protein-protein or protein-ligand interaction | ^36^ |
|  | SOM MAM and LDL receptor 1  (B3EWZ5) | Skeletal organic matrix (SOM) | 8.29 | Asp – 6.3%  Glu – 2.5%  **8.8%** | MAM domain (cd06263) X24;  MAM domain (pfam00629) X3;  P or trefoil or TFF domain (smart00018);  Trefoil (P-type) domain (pfam00088);  Low Density Lipoprotein Receptor Class A domain (cd00112) X8 | Extracellular domain, protein-protein interaction, cell adhesion | ^36^ |
|  | SOM MAM and LDL receptor 2  (B3EWZ6) | Skeletal organic matrix (SOM) | 7.34 | Asp – 6.7%  Glu – 2.8%  **9.5%** | MAM domain (cd06263) X31;  MAM domain (pfam00629) X4;  MAM domain (cl27660);  P or trefoil or TFF domain (smart00018);  Trefoil (P-type) domain (pfam00088);  Low Density Lipoprotein Receptor Class A domain (cd00112) X14;  Low Density Lipoprotein Receptor Class A domain (smart00192) | Extracellular domain, protein-protein interaction, cell adhesion  Extracellular domain, protein-protein interaction, cell adhesion | ^36^ |
|  | Threonine-rich protein  (B3EWZ7) | Skeletal organic matrix (SOM) | 4.01 | Asp – 2.4%  Glu -7.6%  **10.0%** | None |  | ^36^ |
|  | Hephaestin-like protein  (B3EWZ9) | Skeletal organic matrix (SOM) | 5.79 | Asp – 7.6%  Glu – 5.7%  **13.3%** | Cupredoxin superfamily (cl19115) X6 | Inter-molecular electron transfer | ^36^ |
|  | Putative carbonic anhydrase  (B8V7P3) | Skeletal organic matrix (SOM) | 9.79 | Asp – 3.5%  Glu – 4.0%  **7.5%** | Carbonic anhydrase alpha (vertebrate-like) group (cl00012) | Reversible hydration of carbon dioxide to bicarbonate | ^36^ |
|  | CUB and peptidase-containing protein 1  (B8V7S0) | Skeletal organic matrix (SOM) | 9.11 | Asp – 3.4%  Glu – 2.3%  **5.7%** | Trypsin-like serine protease (cd00190);  CUB domain (cd00041);  CUB domain (cl00049) | Serine protease;  Extracellular domain, typically involved in development | ^36^ |
|  | CUB and peptidase-containing protein 2  (B8VIV4) | Skeletal organic matrix (SOM) | 8.81 | Asp – 3.3%  Glu – 3.1%  **6.4%** | Trypsin-like serine protease (cd00190);  Trypsin-like serine protease (cl21584);  CUB domain (cd00041) | Serine protease;  Extracellular domain, typically involved in development | ^36^ |
|  | Cephalotoxin-like protein  (B7W114) | Skeletal organic matrix (SOM) | 9.26 | Asp – 6.1%  Glu – 5.3%  **11.4%** | None |  | ^36^ |
|  | Skeletal aspartic acid-rich protein 1  (B3EWY6) | Skeletal organic matrix (SOM) | 3.96 | Asp - 19.2%  Glu – 5.4%  **24.6%** | None |  | ^36^ |
|  | Skeletal aspartic acid-rich protein 2  (B3EWY8) | Skeletal organic matrix (SOM) | 4.25 | Asp – 20.3%  Glu – 6.4%  **26.7%** | None |  | ^36^ |
|  | Acidic SOMP  (B3EWY7) | Skeletal organic matrix (SOM) | 4.15 | Asp – 9.2%  Glu – 8.1%  **17.3%** | None |  | ^36^ |
|  | Secreted acidic protein 1  (B3EWZ0) | Skeletal organic matrix (SOM) | 3.43 | Asp – 27.4%  Glu – 11.9%  **39.3%** | None |  | ^36^ |
|  | Secreted acidic protein 2  (B3EWZ4) | Skeletal organic matrix (SOM) | 3.28 | Asp – 30.3%  Glu – 3.6%  **33.9%** | None |  | ^36^ |
|  | Glutamic acid-rich protein  (B7W112) | Skeletal organic matrix (SOM) | 3.95 | Asp – 19.0%  Glu – 21.6%  **40.6%** | Midasin, AAA ATPase with vWA domain (cl34967) | Ribosome maturation | ^36^ |
|  | Uncharacterised SOMP 1  (B3EX00) | Skeletal organic matrix (SOM) | 10.97 | Asp – 3.1%  Glu – 2.9%  **6.0%** | None |  | ^36^ |
|  | Uncharacterised SOMP 2  (B7WFQ1) | Skeletal organic matrix (SOM) | 5.96 | Asp – 4.6%  Glu – 5.0%  **9.6%** | None |  | ^36^ |
|  | Uncharacterised SOMP 3  (B8RJM0) | Skeletal organic matrix (SOM) | 7.38 | Asp – 4.2%  Glu – 6.5%  **10.7%** | None |  | ^36^ |
|  | Uncharacterised SOMP 4  (B8UU74) | Skeletal organic matrix (SOM) | 10.01 | Asp – 3.4%  Glu – 4.4%  **7.8%** | None |  | ^36^ |
|  | Uncharacterised SOMP 5  (B8VIU6) | Skeletal organic matrix (SOM) | 9.25 | Asp – 3.1%  Glu – 2.3%  **5.4%** | None |  | ^36^ |
|  | Uncharacterised SOMP 6  (B8VIX3) | Skeletal organic matrix (SOM) | 9.02 | Asp – 2.8%  Glu – 13.8%  **16.6%** | Nup88 super family (cl25737) | Nucleoporin-nuclear membrane tethering | ^36^ |
|  | Uncharacterised SOMP 7  (B8WI85) | Skeletal organic matrix (SOM) | 9.27 | Asp – 4.3%  Glu – 3.8%  **8.1%** | None |  | ^36^ |
|  | Uncharacterised SOMP 8  (B3EWZ2) | Skeletal organic matrix (SOM) | 5.42 | Asp – 4.7%  Glu – 6.1%  **10.8%** | None |  | ^36^ |
|  | Galaxin  (D9IQ16) | Skeletal organic matrix (SOM) | 5.45 | Asp – 4.4%  Glu – 2.7%  **7.1%** | None |  | ^36^ |
|  | Galaxin 2  (B8UU51) | Skeletal organic matrix (SOM) | 8.23 | Asp – 4.0%  Glu – 0.0%  **4.0%** | None |  | ^36^ |

*****Asp – aspartic acid; Glu – glutamic acid; amino acid proportion and theoretical pI values derived from Expasy ProtParam Tool

**References**

1. Skeffington, A.; Fischer, A.; Sviben, S.; Brzezinka, M.; Górka, M.; Bertinetti, L.; Woehle, C.; Huettel, B.; Graf, A.; Scheffel, A., A joint proteomic and genomic investigation provides insights into the mechanism of calcification in coccolithophores. *Nature Communications* **2023,** *14* (1), 3749.

2. Norizuki, M.; Samata, T., Distribution and function of the nacrein-related proteins inferred from structural analysis. *Mar Biotechnol (NY)* **2008,** *10* (3), 234-41.

3. Wang, X.; Song, X.; Wang, T.; Zhu, Q.; Miao, G.; Chen, Y.; Fang, X.; Que, H.; Li, L.; Zhang, G., Evolution and functional analysis of the Pif97 gene of the Pacific oyster Crassostrea gigas. *Current Zoology* **2013,** *59* (1), 109-115.

4. Song, X.; Wang, X.; Li, L.; Zhang, G., Identification two novel nacrein-like proteins involved in the shell formation of the Pacific oyster Crassostrea gigas. *Mol Biol Rep* **2014,** *41* (7), 4273-8.

5. Weiss, I. M.; Gohring, W.; Fritz, M.; Mann, K., Perlustrin, a Haliotis laevigata (abalone) nacre protein, is homologous to the insulin-like growth factor binding protein N-terminal module of vertebrates. *Biochem Biophys Res Commun* **2001,** *285* (2), 244-9.

6. Mann, K.; Weiss, I. M.; Andre, S.; Gabius, H. J.; Fritz, M., The amino-acid sequence of the abalone (Haliotis laevigata) nacre protein perlucin. Detection of a functional C-type lectin domain with galactose/mannose specificity. *Eur J Biochem* **2000,** *267* (16), 5257-64.

7. Shen, X.; Belcher, A. M.; Hansma, P. K.; Stucky, G. D.; Morse, D. E., Molecular cloning and characterization of lustrin A, a matrix protein from shell and pearl nacre of Haliotis rufescens. *J Biol Chem* **1997,** *272* (51), 32472-81.

8. Michenfelder, M.; Fu, G.; Lawrence, C.; Weaver, J. C.; Wustman, B. A.; Taranto, L.; Evans, J. S.; Morse, D. E., Characterization of two molluscan crystal-modulating biomineralization proteins and identification of putative mineral binding domains. *Biopolymers* **2003,** *70* (4), 522-533.

9. Sarashina, I.; Endo, K., Primary structure of a soluble matrix protein of scallop shell; implications for calcium carbonate biomineralization. *Am Mineral* **1998,** *83* (11-12_Part_2), 1510-1515.

10. Fang, D.; Xu, G.; Hu, Y.; Pan, C.; Xie, L.; Zhang, R., Identification of genes directly involved in shell formation and their functions in pearl oyster, Pinctada fucata. *PLoS One* **2011,** *6* (7), e21860.

11. Werner, G. D.; Gemmell, P.; Grosser, S.; Hamer, R.; Shimeld, S. M., Analysis of a deep transcriptome from the mantle tissue of Patella vulgata Linnaeus (Mollusca: Gastropoda: Patellidae) reveals candidate biomineralising genes. *Mar Biotechnol (NY)* **2013,** *15* (2), 230-43.

12. Miyamoto, H.; Miyashita, T.; Okushima, M.; Nakano, S.; Morita, T.; Matsushiro, A., A carbonic anhydrase from the nacreous layer in oyster pearls. *Proc Natl Acad Sci U S A* **1996,** *93* (18), 9657-60.

13. Yu, Z.; Xie, L.; Lee, S.; Zhang, R., A novel carbonic anhydrase from the mantle of the pearl oyster (Pinctada fucata). *Comp Biochem Physiol B Biochem Mol Biol* **2006,** *143* (2), 190-4.

14. Samata, T.; Hayashi, N.; Kono, M.; Hasegawa, K.; Horita, C.; Akera, S., A new matrix protein family related to the nacreous layer formation of Pinctada fucata. *FEBS Lett* **1999,** *462* (1-2), 225-9.

15. Sudo, S.; Fujikawa, T.; Nagakura, T.; Ohkubo, T.; Sakaguchi, K.; Tanaka, M.; Nakashima, K.; Takahashi, T., Structures of mollusc shell framework proteins. *Nature* **1997,** *387* (6633), 563-4.

16. Zhang, Y.; Xie, L.; Meng, Q.; Jiang, T.; Pu, R.; Chen, L.; Zhang, R., A novel matrix protein participating in the nacre framework formation of pearl oyster, Pinctada fucata. *Comp Biochem Physiol B Biochem Mol Biol* **2003,** *135* (3), 565-73.

17. Tsukamoto, D.; Sarashina, I.; Endo, K., Structure and expression of an unusually acidic matrix protein of pearl oyster shells. *Biochemical and Biophysical Research Communications* **2004,** *320* (4), 1175-1180.

18. Suzuki, M.; Murayama, E.; Inoue, H.; Ozaki, N.; Tohse, H.; Kogure, T.; Nagasawa, H., Characterization of Prismalin-14, a novel matrix protein from the prismatic layer of the Japanese pearl oyster (Pinctada fucata). *Biochem J* **2004,** *382* (Pt 1), 205-13.

19. Suzuki, M.; Saruwatari, K.; Kogure, T.; Yamamoto, Y.; Nishimura, T.; Kato, T.; Nagasawa, H., An acidic matrix protein, Pif, is a key macromolecule for nacre formation. *Science* **2009,** *325* (5946), 1388-90.

20. Zhang, C.; Xie, L.; Huang, J.; Liu, X.; Zhang, R., A novel matrix protein family participating in the prismatic layer framework formation of pearl oyster, Pinctada fucata. *Biochem Biophys Res Commun* **2006,** *344* (3), 735-40.

21. Pan, C.; Fang, D.; Xu, G.; Liang, J.; Zhang, G.; Wang, H.; Xie, L.; Zhang, R., A novel acidic matrix protein, PfN44, stabilizes magnesium calcite to inhibit the crystallization of aragonite. *J Biol Chem* **2014,** *289* (5), 2776-87.

22. Liu, H. L.; Liu, S. F.; Ge, Y. J.; Liu, J.; Wang, X. Y.; Xie, L. P.; Zhang, R. Q.; Wang, Z., Identification and characterization of a biomineralization related gene PFMG1 highly expressed in the mantle of Pinctada fucata. *Biochemistry* **2007,** *46* (3), 844-51.

23. Huang, J.; Zhang, C.; Ma, Z.; Xie, L.; Zhang, R., A novel extracellular EF-hand protein involved in the shell formation of pearl oyster. *Biochim Biophys Acta* **2007,** *1770* (7), 1037-44.

24. Yano, M.; Nagai, K.; Morimoto, K.; Miyamoto, H., Shematrin: a family of glycine-rich structural proteins in the shell of the pearl oyster Pinctada fucata. *Comp Biochem Physiol B Biochem Mol Biol* **2006,** *144* (2), 254-62.

25. Yan, Y.; Yang, D.; Yang, X.; Liu, C.; Xie, J.; Zheng, G.; Xie, L.; Zhang, R., A Novel Matrix Protein, PfY2, Functions as a Crucial Macromolecule during Shell Formation. *Sci Rep* **2017,** *7* (1), 6021.

26. Ma, Z.; Huang, J.; Sun, J.; Wang, G.; Li, C.; Xie, L.; Zhang, R., A novel extrapallial fluid protein controls the morphology of nacre lamellae in the pearl oyster, Pinctada fucata. *J Biol Chem* **2007,** *282* (32), 23253-63.

27. Xie, J.; Liang, J.; Sun, J.; Gao, J.; Zhang, S.; Liu, Y.; Xie, L.; Zhang, R., Influence of the Extrapallial Fluid of Pinctada fucata on the Crystallization of Calcium Carbonate and Shell Biomineralization. *Crystal Growth & Design* **2016,** *16* (2), 672-680.

28. Kono, M.; Hayashi, N.; Samata, T., Molecular Mechanism of the Nacreous Layer Formation in Pinctada maxima. *Biochemical and Biophysical Research Communications* **2000,** *269* (1), 213-218.

29. Wang, Y.; Xia, J.; Tang, R.; Yu, D., Cloning and characterization of nacre-related genes in silver-lip pearl oyster Pinctada maxima. *Journal of Shanghai Ocean University* **2011,** *20* (1), 8-14.

30. Isowa, Y.; Sarashina, I.; Setiamarga, D. H.; Endo, K., A comparative study of the shell matrix protein aspein in pterioid bivalves. *J Mol Evol* **2012,** *75* (1-2), 11-8.

31. Suzuki, M.; Iwashima, A.; Kimura, M.; Kogure, T.; Nagasawa, H., The molecular evolution of the pif family proteins in various species of mollusks. *Mar Biotechnol (NY)* **2013,** *15* (2), 145-58.

32. McDougall, C.; Aguilera, F.; Degnan, B. M., Rapid evolution of pearl oyster shell matrix proteins with repetitive, low-complexity domains. *J R Soc Interface* **2013,** *10* (82), 20130041.

33. Joubert, C.; Piquemal, D.; Marie, B.; Manchon, L.; Pierrat, F.; Zanella-Cleon, I.; Cochennec-Laureau, N.; Gueguen, Y.; Montagnani, C., Transcriptome and proteome analysis of Pinctada margaritifera calcifying mantle and shell: focus on biomineralization. *BMC Genomics* **2010,** *11*, 613.

34. Marin, F.; Corstjens, P.; de Gaulejac, B.; de Vrind-De Jong, E.; Westbroek, P., Mucins and molluscan calcification. Molecular characterization of mucoperlin, a novel mucin-like protein from the nacreous shell layer of the fan mussel Pinna nobilis (Bivalvia, pteriomorphia). *J Biol Chem* **2000,** *275* (27), 20667-75.

35. Drake, J. L.; Mass, T.; Haramaty, L.; Zelzion, E.; Bhattacharya, D.; Falkowski, P. G., Proteomic analysis of skeletal organic matrix from the stony coral Stylophora pistillata. *Proceedings of the National Academy of Sciences* **2013,** *110* (10), 3788-3793.

36. Ramos-Silva, P.; Kaandorp, J.; Huisman, L.; Marie, B.; Zanella-Cléon, I.; Guichard, N.; Miller, D. J.; Marin, F., The Skeletal Proteome of the Coral Acropora millepora: The Evolution of Calcification by Co-Option and Domain Shuffling. *Molecular Biology and Evolution* **2013,** *30* (9), 2099-2112.
