# Supplementary material for: Exploring proteins within the coccolith matrix
Source: Sci Rep. 2024 Dec 30;14:31821. doi: 10.1038/s41598-024-83052-9 (PMC11685980; doi:10.1038/s41598-024-83052-9)
Supplement: Supplementary file 2 — Supplementary Material 2 [file 41598_2024_83052_MOESM2_ESM.docx]

**Exploring proteins associated with the coccolith matrix**

Craig J. Dedman,^1*†^ Nishant Chauhan,^1*^ Alba González-Lanchas,^1^ Chloë Baldreki,^2^ Adam A. Dowle,^2^ Tony R. Larson,^2^ Renee B. Y. Lee,^3^ Rosalind E. M. Rickaby^1^

^1^Department of Earth Sciences, University of Oxford, South Parks Rd, Oxford, OX1 3AN, UK

^2^Bioscience Technology Facility, Department of Biology, University of York, UK

^3^School of Biological Sciences, University of Reading, Whiteknights, Reading, RG6 6UB, UK

^*^Joint first author

^†^corresponding author: Craig J. Dedman (craig.dedman@earth.ox.ac.uk)

**Supplementary Information**

**Section SI.1: Summary of identified proteins**

1. RCC1242 Non-calcifying *Gephyrocapsa huxleyi* and calcium carbonate control

**Table SI.1.** Proteins identified in coccoliths derived from RCC1242 *G. huxleyi* & CaCO_3_ control

| **Protein group** | **No. of proteins in group** | **Max. protein coverage (%)** | **Conserved domain/**  **Protein family** | **Typical function** | **Signal peptide present?** |
| --- | --- | --- | --- | --- | --- |
| 60 | 1 | 9 | Histone H4/H3 | Chromatin structure | No |
| 73 | 7 | 3 | Heat shock protein 70 family | Cytoskeleton | No |
| 75 | 3 | 2 | Intermembrane transport protein PqiA-lik | Intermembrane transport | Yes (3/3) |

1. RCC1731 *Gephyrocapsa huxleyi*

**Table SI.2.** Proteins identified in coccoliths derived from RCC1731 *G. huxleyi*

| **Protein group** | **No. of proteins in group** | **Max. protein coverage (%)** | **Conserved domain/**  **Protein family** | **Typical function** | **Signal peptide present?** |
| --- | --- | --- | --- | --- | --- |
| 62 | 12 | 13 | 14-3-3 protein | Protein localisation; Cell signalling | No |
| 50 | 15 | 25 | Actin family | Cytoskeleton | Yes (1/15) |
| 79 | 2 | 1 | Chromosome segregation ATPase Smc; Acyl transferase domain | Cell division; Lipid metabolism | No |
| 51 | 1 | 19 | Cupredoxin superfamily | Copper ion binding; Electron transport | Yes (1/1) |
| 58 | 1 | 1 | Various DNA-interacting | Transcription | No |
| 43 | 1 | 20 | Pentapeptide-repeat | Unknown | Yes (1/1) |
| 52 | 3 | 28 | Pentapeptide-repeat | Unknown | No |
| 59 | 2 | 7 | Pentapeptide-repeat | Unknown | Yes (2/2) |
| 64 | 1 | 5 | Pentapeptide-repeat | Unknown | No |
| 36 | 2 | 21 | None | - | Yes (2/2) |
| 39 | 1 | 20 | None | - | Yes (1/1) |
| 40 | 7 | 30 | None | - | Yes (7/7) |
| 49 | 4 | 19 | None | - | Yes (1/4) |

1. RCC1314 *Gephyrocapsa oceanica*

**Table SI.3.** Proteins identified in coccoliths derived from RCC1314 *G. oceanica*

| **Protein group** | **No. of proteins in group** | **Max. protein coverage (%)** | **Conserved domain/**  **Protein family** | **Typical function** | **Signal peptide present?** |
| --- | --- | --- | --- | --- | --- |
| 112 | 2 | 8 | 14-3-3 protein | Protein localisation; Cell signalling | No |
| 92 | 2 | 27 | SAM (Sterile alpha motif) | Protein binding; Cell signalling | Yes (2/2) |
| 109 | 1 | 16 | Ubiquitin and ubiquitin-like | Protein ubiquitination | No |
| 135 | 1 | 20 | Ubiquitin and ubiquitin-like | Protein ubiquitination | Yes (1/1) |
| 134 | 1 | 4 | Plant receptor-like serine/threonine kinase | Protein binding | No |
| 54 | 1 | 32 | Protein disulfide isomerase | Protein folding; Stress response | Yes (1/1) |
| 111 | 1 | 16 | Cyclophilin | Protein peptidyl-prolyl isomerization | Yes (1/1) |
| 138 | 2 | 16 | FKBP-type Peptidyl-prolyl cis-trans Isomerase | Protein peptidyl-prolyl isomerization | Yes (2/2) |
| 169 | 1 | 5 | Heat shock protein Hsp90 family | Chaperone protein | No |
| 106 | 1 | 15 | Tic22-like | Protein transport | Yes (1/1) |
| 26 | 3 | 12 | Subtilisin-like serine protease | Proteolysis | Yes (3/3) |
| 43 | 1 | 21 | C1 Peptidase family | Proteolysis | Yes (1/1) |
| 93 | 3 | 15 | Peptidase C13 family | Proteolysis | Yes (2/3) |
| 104 | 1 | 6 | Glutamyl Endopeptidase | Proteolysis | No |
| 95 | 2 | 37 | Cystatin-like domain | Endopeptidase inhibitor activity | Yes (2/2) |
| 84 | 1 | 23 | Ran GTPase-activating protein | mRNA processing and transport | Yes (1/1) |
| 131 | 1 | 7 | 50S ribosomal subunit-associated GTPase HflX | Translation | Yes (1/1) |
| 25 | 1 | 58 | PAN/APPLE-like domain | Carbohydrate binding | No |
| 38 | 1 | 21 | Glycosyl transferase, family 8 | Carbohydrate metabolism | No |
| 114 | 1 | 6 | Glycosyl Hydrolase Family 51 | Carbohydrate metabolism | Yes (1/1) |
| 130 | 3 | 8 | Glycoside hydrolase, family 27 | Carbohydrate metabolism | No |
| 139 | 2 | 5 | Glycoside hydrolase, family 27 | Carbohydrate metabolism | Yes (2/2) |
| 59 | 1 | 44 | Cupredoxin superfamily | Copper ion binding; Electron transport | No |
| 100 | 1 | 6 | Fibronectin Type II domain | Cell adhesion | No |
| 170 | 1 | 10 | Pistil-specific extensin-like protein | Cell wall protein | No |
| 133 | 1 | 6 | FAD-linked Oxidoreductases in Biosynthetic Pathways | Biosynthesis | No |
| 122 | 3 | 7 | ATP synthase CF1 beta subunit | ATP Synthesis | No |
| 60 | 1 | 30 | P-loop containing Nucleoside Triphosphate Hydrolases | Multiple | Yes (1/1) |
| 107 | 1 | 9 | Methyltransferase domain | Methyltransferase activity | Yes (1/1) |
| 129 | 5 | 12 | Probable methyltransferase-like protein 24 | Methyltransferase activity | Yes (3/5) |
| 184 | 3 | 11 | Methyltransferase (Class A) | Methyltransferase activity | No |
| 11 | 1 | 51 | Pentapeptide-repeat | Unknown | Yes (1/1) |
| 30 | 1 | 40 | Pentapeptide-repeat | Unknown | No |
| 36 | 1 | 33 | Pentapeptide-repeat | Unknown | Yes (1/1) |
| 39 | 1 | 36 | Pentapeptide-repeat | Unknown | Yes (1/1) |
| 42 | 1 | 41 | Pentapeptide-repeat | Unknown | No |
| 46 | 1 | 22 | Pentapeptide-repeat | Unknown | No |
| 47 | 1 | 33 | Pentapeptide-repeat | Unknown | No |
| 48 | 1 | 37 | Pentapeptide-repeat | Unknown | No |
| 51 | 1 | 34 | Pentapeptide-repeat | Unknown | Yes (1/1) |
| 52 | 1 | 29 | Pentapeptide-repeat | Unknown | No |
| 53 | 1 | 16 | Pentapeptide-repeat | Unknown | No |
| 56 | 3 | 15 | Pentapeptide-repeat | Unknown | Yes (3/3) |
| 62 | 1 | 18 | Pentapeptide-repeat | Unknown | No |
| 65 | 1 | 22 | Pentapeptide-repeat | Unknown | No |
| 68 | 1 | 19 | Pentapeptide-repeat | Unknown | No |
| 70 | 2 | 24 | Pentapeptide-repeat | Unknown | No |
| 74 | 2 | 22 | Pentapeptide-repeat | Unknown | No |
| 76 | 2 | 14 | Pentapeptide-repeat | Unknown | Yes (2/2) |
| 83 | 1 | 21 | Pentapeptide-repeat | Unknown | Yes (1/1) |
| 87 | 2 | 25 | Pentapeptide-repeat | Unknown | No |
| 89 | 1 | 11 | Pentapeptide-repeat | Unknown | No |
| 91 | 1 | 11 | Pentapeptide-repeat | Unknown | Yes (1/1) |
| 94 | 1 | 28 | Pentapeptide-repeat | Unknown | No |
| 101 | 2 | 17 | Pentapeptide-repeat | Unknown | No |
| 103 | 1 | 12 | Pentapeptide-repeat | Unknown | Yes (1/1) |
| 105 | 1 | 12 | Pentapeptide-repeat | Unknown | No |
| 113 | 2 | 13 | Pentapeptide-repeat | Unknown | No |
| 116 | 1 | 25 | Pentapeptide-repeat | Unknown | No |
| 120 | 1 | 11 | Pentapeptide-repeat | Unknown | No |
| 137 | 10 | 9 | Pentapeptide-repeat | Unknown | Yes (10/10) |
| 57 | 2 | 17 | None | - | Yes (2/2) |
| 58 | 1 | 17 | None | - | Yes (1/1) |
| 63 | 1 | 23 | None | - | No |
| 64 | 1 | 26 | None | - | No |
| 69 | 1 | 19 | None | - | Yes (1/1) |
| 75 | 2 | 24 | None | - | No |
| 77 | 1 | 21 | None | - | Yes (1/1) |
| 78 | 1 | 17 | None | - | Yes (1/1) |
| 79 | 3 | 13 | None | - | Yes (3/3) |
| 80 | 1 | 16 | None | - | No |
| 85 | 2 | 10 | None | - | No |
| 88 | 1 | 30 | None | - | No |
| 96 | 1 | 8 | None | - | Yes (1/1) |
| 98 | 2 | 11 | None | - | Yes (1/2) |
| 110 | 2 | 6 | None | - | Yes (2/2) |
| 118 | 1 | 18 | None | - | No |
| 128 | 2 | 11 | None | - | No |
| 171 | 1 | 23 | None | - | No |

1. RCC1198 *Coccolithus braarudii*

**Table SI.4.** Proteins identified in coccoliths derived from RCC1198 *C. braarudii*

| **Protein group** | **No. of proteins in group** | **Max. protein coverage (%)** | **Conserved domain/**  **Protein family** | **Typical function** | **Signal peptide present?** |
| --- | --- | --- | --- | --- | --- |
| 112 | 3 | 7 | 14-3-3 protein | Protein localisation; Cell signalling | No |
| 108 | 1 | 32 | Ubiquitin and ubiquitin-like | Protein ubiquitination | No |
| 163 | 1 | 6 | Protein TIC110 | Protein transport | Yes (1/1) |
| 137 | 2 | 11 | Proteasome subunit alpha | Proteolysis | No |
| 92 | 1 | 24 | Histone H2A/H2B/H3 domain | Chromatin structure | No |
| 97 | 1 | 21 | histone-fold domain found in histone H2B | Chromatin structure | No |
| 178 | 1 | 7 | DEAD-box helicase superfamily | Helicase activity | No |
| 194 | 1 | 22 | helicase superfamily | Helicase activity | No |
| 197 | 1 | 6 | RRM superfamily | RNA binding | No |
| 210 | 1 | 17 | DEAD-box protein superfamily | RNA binding | No |
| 136 | 1 | 31 | 40S ribosomal protein S14 | Translation | No |
| 192 | 1 | 10 | 40S ribosomal protein S14 | Translation | No |
| 144 | 1 | 8 | Chromosome segregation ATPase Smc | Cell division | No |
| 172 | 1 | 6 | Kinesin-like protein | Cytoskeleton | No |
| 101 | 1 | 10 | FoF1-type ATP synthase, beta subunit | ATP synthesis | No |
| 111 | 1 | 9 | F0F1 ATP synthase subunit alpha | ATP synthesis | No |
| 105 | 1 | 22 | Mitochondrial carrier protein | ADP/ATP transporter | Yes (1/1) |
| 113 | 1 | 8 | Glyceraldehyde-3-phosphate dehydrogenase | Glycolysis/Gluconeogenesis | No |
| 146 | 1 | 13 | Glyceraldehyde-3-phosphate dehydrogenase | Glycolysis/Gluconeogenesis | No |
| 123 | 1 | 7 | Malate dehydrogenase | TCA cycle | No |
| 120 | 1 | 7 | Sulfotransferase family | S metabolism | No |
| 173 | 1 | 8 | Abhydrolase_1 | Hydrolase activity | No |
| 126 | 1 | 6 | P-loop containing Nucleoside Triphosphate Hydrolases | Various | No |
| 115 | 1 | 7 | Pentapeptide-repeat | Unknown | Yes (1/1) |
| 118 | 1 | 5 | Pentapeptide-repeat | Unknown | No |
| 121 | 1 | 7 | None | - | No |
| 164 | 1 | 24 | None | - | No |
| 169 | 1 | 8 | None | - | No |
| 177 | 1 | 22 | None | - | No |

1. Overlapping proteins in RCC1731 *G. huxleyi* with Skeffington *et al.* (2023) COPROs dataset

**Table SI.5.** *G. huxleyi* coccolith proteins identified in multiple studies

| **Protein group** | **Accession** | **Protein coverage (%)** | **Conserved domain/**  **Protein family** | **Typical function** | **Signal peptide present?** |
| --- | --- | --- | --- | --- | --- |
| 36 | EhG21537.1 | 21 | None | - | Yes |
| 39 | EhG41650.1 | 20 | None | - | Yes |
| 40 | EhG17242.1 | 22 | None | - | Yes |
| 40 | EhG17242.7 | 30 | None | - | Yes |
| 43 | EhG5157.1 | 20 | Pentapeptide-repeat | Unknown | Yes |
| 59 | EhG40669.2 | 6 | Pentapeptide-repeat | Unknown | Yes |

**Section SI.2. BLASTp sequence alignment & similarity search**

The BLASTp sequence alignment tool was used to assess similarity between identified proteins identified in each respective species. Data was filtered to include proteins with >90% similarity. Results are summarised in Tables SI.6-8, below.
